# Protein-Based Nanomaterials for Cancer Therapy: A Comparative and Translational Perspective

**DOI:** 10.3390/pharmaceutics18070831

**Published:** 2026-07-07

**Authors:** Juan Gonzalez-Valdivieso, Javier Gutiérrez, Jonathan Alexander Vásquez Calero, Sara Escalera-Anzola, Raquel Muñoz, Francisco Javier Arias, M. Ángeles Rojo, Alessandra Girotti

**Affiliations:** 1 Smart Devices for NanoMedicine, University of Valladolid, LUCIA Building, 47011 Valladolid, Spain; juan.gonzalez.valdivieso@uva.es (J.G.-V.); javier.gutierrez.reguera@uva.es (J.G.); jonathanalexander.vasquez@estudiantes.uva.es (J.A.V.C.); sara.escalera@uva.es (S.E.-A.); raquel.munoz.martinez@uva.es (R.M.); jarias@uva.es (F.J.A.); 2 Instituto de Investigación Biosanitaria de Valladolid (IBioVALL), 47010 Valladolid, Spain; 3 Unidad de Excelencia Instituto de Biomedicina y Genética Molecular (IBGM), Centro Superior de Investigaciones Científicas (CSIC), Universidad de Valladolid, 47003 Valladolid, Spain; 4 Department of Experimental Sciences, Miguel de Cervantes European University, 47012 Valladolid, Spain

**Keywords:** protein nanomaterials, cancer therapies, nanomedical devices

## Abstract

Over the past decade, the use of nanomaterials and nanomedical devices has been increasingly explored for cancer treatment. Although the outcomes of conventional therapies have improved patient survival, these approaches still present important limitations for some types of cancer and metastasis. Challenges such as poor drug accumulation in solid tumors and lack of specificity and selectivity can be addressed through alternative nanomedicine-based treatments. Among the wide range of nanoplatforms whose composition and shape have been designed for cancer treatment, this review focuses specifically on those based on natural proteins, including advanced carriers and engineered proteins bearing active targeting and/or therapeutic agents. The objective of this review is to provide a comparative and translational analysis of protein-based nanomaterials for cancer therapy, highlighting their unique characteristics, such as biocompatibility, biodegradability, and the ability to integrate bioactive peptides that can trigger or respond to tumor-specific or altered physiological stimuli. Several protein-based nanomedical devices have been developed for theranostic applications, demonstrating enhanced performance in tumor imaging and cancer treatment. This review introduces a structured analytical framework that classifies protein-based nanomaterials according to their biological origin, functional design, and clinical readiness, enabling systematic evaluation across platforms. Rather than providing a descriptive overview, this work offers a structured comparative analysis of protein-based nanomaterials, highlighting design trade-offs, translational challenges, and factors influencing clinical applicability.

## 1. Introduction

Standard anticancer treatments based on surgery, radiotherapy and chemotherapy show a survival benefit in patients with localized tumors; however, they still present some significant challenges [1,2]. Beyond poor drug accumulation in some solid tumors, chemotherapy suffers from limited specificity and selectivity, leading to severe toxicity in organs such as the heart, nervous system, bone marrow, and gastrointestinal tract [3,4].

Cancer nanomedicine presents potential advantages over conventional therapies through different therapeutic delivery systems (e.g., drug depots [5], microspheres [6], nanocarriers [7]). Among the nanodevices currently being applied, the use and development of organic, inorganic and polymeric nanocarriers [8] can be highlighted [9,10,11]. Although nanomedicines are already used in clinics and numerous clinical trials are in progress, their clinical translation remains limited; therefore, strategies to overcome these difficulties are required [1,12,13,14].

An integrated vision of cancer is essential for the development of effective therapy with translational potential. Tumors are dynamic and complex systems in which cancer cells interact with stromal components, immune cells, and the extracellular matrix (ECM), forming the tumor microenvironment (TME) [15,16,17]. This microenvironment evolves to support tumor growth, invasion, metastases and resistance to therapy [18,19,20].

The structural and functional heterogeneity of the TME—including abnormal vasculature [21,22], ECM remodeling, and variable immune infiltration [23]—creates significant barriers to treatment. These features lead to elevated interstitial pressure [24], hypoxia, and acidic conditions [25], which further limit drug distribution and contribute to immune evasion and therapeutic resistance [26,27]. Consequently, the TME presents dynamic spatio-temporal barriers that hinder the penetration, homogeneous distribution, and efficacy of systemically administered nanomedicines. Although the enhanced permeability and retention (EPR) effect has been widely used to explain nanoparticle accumulation in tumors, its relevance is highly variable and context-dependent [28,29]. Passive targeting, which relies on these TME-associated features, is therefore inherently limited by interpatient heterogeneity and the lack of robust biomarkers for patient stratification. In contrast, active targeting strategies based on ligand-functionalized nanocarriers aim to enhance cellular specificity and internalization; however, their effectiveness remains contingent upon adequate extravasation and is often compromised by protein corona formation, which can mask targeting ligands and alter nanocarrier–cell interactions. Together, these limitations contribute to the persistent gap between preclinical success and clinical efficacy, underscoring the need for stratified nanomedicine approaches that integrate patient-specific TME characteristics, systemic biological variability, and nanocarrier design. Passive and active targeting are not independent strategies but interdependent processes: passive targeting dictates tumor access, whereas active targeting refines cellular specificity. Their integration, rather than their isolated optimization, is therefore essential for improving the performance of protein nanocarriers [30].

In addition, the optimization of nanocarrier physicochemical properties has been pursued to modulate opsonization and subsequent clearance by the mononuclear phagocyte system, thus increasing their systemic availability. Upon exposure to biological fluids, nanocarriers acquire a new biological identity through protein corona formation, composed of opsonins and dysopsonins, which governs their interactions with cells and tissues and ultimately determines their biological fate [31,32]. Therefore, nanocarrier design must explicitly account for this dynamic interface. In this context, biomimetic strategies aim to modulate corona composition, promoting the selective adsorption of proteins that enhance targeting, prolong circulation, and improve cellular uptake.

Protein-based nanocarriers deserve particular consideration, as their intrinsic biochemical cues can influence corona composition and favor more specific protein–protein interactions, potentially improving biocompatibility and reducing nonspecific opsonization [27,32,33]. However, they also present limitations, including conformational instability, possible denaturation exposing cryptic epitopes, and variability in their in vivo behavior, which complicate precise control over their biological identity [27,33,34,35,36]. Surface functionalization with specific ligands permits selective targeting of circulating cancer cells and metastatic niches [37,38,39]. Drug-release behavior remains another key design parameter [27,40], and in this regard, sustained release kinetics in nanomedicines have enabled the development of stimulus-responsive targeted nanocarriers. These features confer a therapeutic advantage when using protein-based nanodevices, particularly those constructed from nanomaterials responsive to specific stimuli of target tissues [40]. This biological and structural complexity underscores the strategic value of advanced nanomaterials. Their tunable physicochemical properties, capacity for targeted delivery, and ability to respond to TME-specific stimuli (such as hypoxia, acidosis, or mechanical stiffness) position nanomaterials—especially protein-based nanomaterials—as powerful tools for overcoming heterogeneous barriers of the TME and enhance therapeutic precision and efficacy [5,27]. Compared to conventional synthetic nanocarriers, protein-based systems offer unique advantages derived from their biological origin, including inherent biocompatibility, structural precision, and the possibility of genetic encoding.

The use of appropriate preclinical animal models and the prioritization of overall survival as an endpoint instead of tumor response are key for therapeutic development and clinical translation [41,42].

A wide range of nanomaterial-based strategies is being explored to overcome tumor-associated barriers, including combination therapies, nucleic acid delivery systems, and nanovaccines, all aimed at enhancing therapeutic efficacy and overcoming drug resistance [27,29]. Theranostic approaches that integrate imaging and therapy are also being developed to monitor biodistribution and treatment response in real time.

Despite these advances, the clinical translation of nanomedicine remains limited due to the complexity of biological interactions, tumor heterogeneity, and challenges in reproducibility, scalability, and regulatory approval [26,27]. These limitations highlight the need for more precisely engineered and biologically compatible nanocarriers [43].

Protein-based nanomaterials can be broadly categorized into endogenous proteins, structurally derived biomaterials, recombinant and engineered systems, and virus-derived architectures. Each of these classes presents distinct advantages and limitations in terms of biological behavior, engineering flexibility, and translational potential. Importantly, although a variety of protein-based nanocarriers have been extensively reviewed, these studies have largely focused on material properties and specific applications rather than providing an integrative framework comparing manufacturability, in vivo performance, and clinical readiness across platforms [44,45,46].

Building on this classification, in the present review, we highlight recent advances in the use of proteins and polypeptides as functional materials for the construction of biodevices and nanomedicines. We place particular emphasis on nanomaterials derived from natural proteins, which have been employed for decades in biomedical applications and have recently undergone significant technological evolution. These nanomaterials originate from diverse protein sources, including elastin, silk fibroin, albumin, ferritin, transferrin, apoferritin, encapsulin and Virus-like particles (VLPs). These biomolecular materials offer unique advantages, including intrinsic biocompatibility, biodegradability, and the ability to be genetically or chemically engineered to display targeting ligands, therapeutic moieties, or stimuli-responsive domains [47]. On this basis, a broad range of biodegradable cancer drug-delivery nanocarriers has been fabricated from natural and recombinant proteins/polypeptides. Depending on the source and chemical modification, these systems can be engineered into nanoparticles, nanofibers, micelles, or hydrogels, with controllable size, charge, and loading capacity. Such properties enable efficient encapsulation or conjugation of chemotherapeutic agents, improved circulation stability, and stimuli-responsive release profiles triggered by pH, redox gradients, or enzymatic activity within the tumor microenvironment [48,49]. In addition, protein–inorganic nanohybrids have emerged as an important class of multifunctional platforms. Incorporation of metallic or inorganic components (e.g., gold, silica, iron oxide, quantum dots) provides mechanical reinforcement, magnetic or optical functionality, and enhanced structural stability. These hybrid systems are being explored for targeted drug delivery, imaging, and theranostic applications, allowing for simultaneous cargo transport, real-time tracking, and localized therapeutic action, such as photothermal or photodynamic effects. The combination of protein-based scaffolds with inorganic nanostructures therefore offers a versatile route to design multifunctional nanomaterials for precision cancer therapy [50].

Previous reviews have mainly categorized protein-based nanocarriers according to material type or multifunctionality, often focusing on physicochemical design and proof-of-concept performance. However, comparatively less attention has been given to translational aspects, such as clinical readiness, manufacturability, scalability, and in vivo performance, which are critical for the successful development of nanomedicines [13,45,51].

In this context, the present review adopts a translationally oriented framework in which protein-based nanomaterials are systematically examined according to parameters relevant to their clinical application. Rather than providing an exhaustive list of systems, this review emphasizes representative protein platforms with demonstrated performance, with the aim of identifying common design principles, key translational bottlenecks, and factors that may influence their progression toward clinical use. By organizing the discussion around these criteria, this work provides a complementary and application-focused perspective to the existing literature, enabling a more direct comparison of protein-based nanomaterials in terms of their translational potential. On this basis, the present review is structured to first examine the biological properties and safety considerations of representative protein-based nanomaterials, followed by their application in theranostic systems and drug delivery platforms. This organization aims to provide a comparative and translational perspective, highlighting key design strategies, limitations, and factors influencing their progression toward clinical application.

## 2. Biological Properties and Safety Considerations of Protein-Based Nanomaterials

Protein-based nanomaterials constitute a class of biomaterials characterized by structural precision, intrinsic biological functionality, and high biocompatibility. Unlike conventional synthetic polymers, these systems are derived from or inspired by naturally occurring proteins, enabling specific molecular recognition, controlled self-assembly, and responsiveness to physiological environments. In cancer therapy, their relevance extends beyond drug delivery efficiency to include targeting specificity, biodegradability, and interaction with biological pathways. Protein-based nanomaterials can be classified into: (i) endogenous proteins (albumin, transferrin, ferritin), (ii) structurally derived biomaterials (silk fibroin), (iii) recombinant and engineered systems (ELPs, encapsulins), and (iv) virus-derived architectures (VLPs). In addition, hybrid systems such as protein–polymer conjugates and membrane-coated nanocarriers have emerged, integrating biological functionality with synthetic tunability and expanding the design space of these platforms.

### 2.1. Albumin: Biological Properties and Safety Considerations

Serum albumin is the most abundant plasma protein [52]. The use of albumin nanoparticles in clinical applications is supported by their physicochemical properties such as biodegradability, low toxicity, low immunogenicity, and ease of preparation. In addition, albumin functions as an extracellular antioxidant, contributing to protection against oxidative stress [52].

From a drug delivery perspective, albumin can undergo conformational changes that facilitate escape from lysosomal and endosomal degradation, which is advantageous for intracellular delivery. The combination of these features supports its application as a carrier by enabling ligand binding and targeted delivery [53,54]. Furthermore, its chemical structure enables interactions with both hydrophobic and hydrophilic therapeutic agents, while protecting them from degradation and improving pharmacokinetics. With regard to immunogenicity, bovine serum albumin (BSA) is a xenogenic protein and may induce immune responses, whereas human serum albumin (HSA) is endogenous and generally considered non-immunogenic in humans, making it the preferred option for clinical applications [55]. Recent preclinical advances have highlighted the versatility of albumin-based systems and their potential across multiple therapeutic areas. These include the development of dual-targeting strategies (e.g., tumor-associated receptors such as mannose receptors), combination therapies, novel drug release mechanisms, and applications in brachytherapy and photodynamic therapy. Additional efforts have focused on the incorporation of chemoresistant agents, as well as the use of biocompatible crosslinkers and formulation components. Beyond oncology, albumin-based platforms are being explored in areas such as metabolic disorders, cardiovascular applications, infectious diseases, and theranostics. Despite these developments, the clinical translation of such systems remains limited, as the progression from preclinical research to regulatory approval is typically prolonged and may be further constrained by challenges related to reproducibility, scalability of manufacturing processes, incomplete characterization of complex formulations, and the lack of well-established regulatory pathways for certain designs. Current clinical efforts are largely centered on expanding the indications of approved formulations, such as Abraxane^®^, while other potential applications remain less extensively investigated [56]. In this context, albumin, as an endogenous circulatory protein with a long history of use in pharmaceutical and medical device applications, represents a well-established platform that supports further translational development [54]. There is increasing interest in extending albumin-based technologies toward applications in the circulatory and lymphatic systems, while also advancing the clinical translation of emerging formulations [55]. Owing to its intrinsic biological properties and favorable clinical profile, albumin-based systems may offer potential for the treatment of diseases affecting these systems, including leukemia and lymphatic malignancies, as well as blood-borne diseases such as hepatitis, human immunodeficiency virus (HIV), malaria, and Ebola virus infection [55,57]. To date, four albumin-based nanomedicines prepared using Nab-technology have been evaluated in clinical trials as potential cancer therapeutics: Abraxane^®^ nab-paclitaxel (ABI-007 at First Line Therapy of Patients With Stage IV Breast Cancer), Fyarro^®^ nab-rapamycin (ABI-009 in Trial in Patients With Advanced Non-hematologic Malignancies), nab-docetaxel (ABI-008 in Trial in Patients With Hormone-refractory Prostate Cancer and in Patients With Metastatic Breast Cancer), and nab-thiocolchicine dimer (ABI-011 in Patients With Advanced Solid Tumors or Lymphomas) [53].

### 2.2. Silk Fibroin: Biological Properties and Safety Considerations

Silk fibroin is a natural hydrophilic glycoprotein derived from silkworm silk and primarily composed of amino acids such as alanine, glycine, and serine, which contribute to hydrogen bond formation and determine its mechanical strength and stability [58]. It exhibits favorable characteristics, including ease of modification, high biocompatibility, tunable biodegradability, low inflammatory response, and good permeability.

From an immunological standpoint, adverse responses, including hypersensitivity reactions, are mainly associated with sericin residues present in raw silk. Once these components are removed, silk fibroin shows biocompatibility comparable to that of synthetic polymers such as PLA, PGA, and PLGA, as well as other natural biomaterials. In addition, preclinical studies suggest potential intrinsic anti-inflammatory activity [59].

Regarding biodegradation, silk fibroin is considered biodegradable provided that its degradation products do not trigger immune responses. Its degradation is a complex process influenced by physical, chemical, and biological factors, and it is generally classified as an enzymatically degradable polymer [60].

Silk fibroin is considered a promising biomaterial due to its favorable safety profile, established use in biomedical applications, and versatility in processing [60]. Both native and recombinant silk fibroin systems have been explored, with recombinant approaches offering improved control over sequence definition and batch-to-batch consistency, thereby facilitating standardization. However, native silk fibroin remains more extensively studied and more readily scalable. The clinical translation of silk fibroin as a drug delivery platform is still limited by variability in raw material sources and processing, together with limited standardization, which impact large-scale manufacturing and reproducibility [60,61]. In addition, aspects such as degradation behavior, long-term safety, and potential immunogenicity require further systematic evaluation [61]. From a translational perspective, the lower price of native silk may explain its predominance in current clinical products over recombinant silk fibroin. However, the use of harsh chemical isolation (i.e., lithium bromide or ionic liquids) during the extraction process causes random protein fragmentation and high polydispersity. This, in turn, makes it difficult to reproduce mechanical properties across different batches. A key requirement when developing silk-based biomaterials for clinical translation remains in their biocompatibility and immunological clearance. Native silk contains sericin, a gummy protein wrapper able to trigger severe hypersensitivity reactions. Therefore, ensuring complete degumming is a critical quality control milestone during manufacturing. On the other hand, recombinant silk fibroin avoids sericin contamination but may introduce lipopolysaccharide (LPS) endotoxins. Thus, an additional expensive purification step, such as polymyxin B chromatography, is needed to satisfy the clinical endotoxin limits (≤0.5 EU/mL) [61,62].

These considerations are supported by preclinical studies, which show enzyme-dependent degradation, low immunogenicity and in vivo remodeling, while degradation of silk nanoparticles has been linked to lysosomal enzymatic activity. Consistent with this, recombinant systems may improve sequence definition and material uniformity. Nevertheless, native Bombyx mori silk fibroin remains the most widely studied source for biomedical and controlled drug-delivery applications [63]. More specifically in oncology drug delivery, clinical translation remains limited: no anti-tumor nanomedicine using silk fibroin as the drug carrier has yet reached the market, and major barriers include raw-material heterogeneity and process-dependent variations in sequence, morphology, and manufacturing, together with the absence of pharmacopeial standards, limited industrial-scale production, and reproducibility issues across studies [64]. Available clinical data are still preliminary and mostly outside oncology drug delivery: a two-patient pilot trial using silk fibroin/collagen scaffold plus autologous chondrocytes for knee osteochondral defects improved IKDC scores and reduced pain from 8 to 0 after one year, while a 50-patient randomized single-blind trial found that a silk fibroin wound dressing reduced severe erythema from 20.8% with Steri-Strips to 0% and reduced wound separation from 30.2% to 9.3% [65,66]. Overall, broader clinical implementation will require improved standardization, scalable manufacturing, robust physicochemical characterization, comprehensive safety evaluation, and direct comparisons between native and recombinant formats [59].

### 2.3. Elastin-like Polypeptides: Biological Properties and Safety Considerations

Elastin-like polypeptides (ELPs) are recombinant biopolymers derived from elastin sequences, which confer elasticity to tissues such as arteries, lungs, and skin [67]. These materials consist of repeating pentapeptides units such as (Val-Pro-Gly-X-Gly), where X can be any amino acid except proline [68], and exhibit thermosensitive phase behavior. Below a transition temperature, ELPs remain soluble, whereas above it they form an insoluble coacervate phase [69], enabling control over solubility, self-assembly, and biological interactions.

In terms of degradation and biocompatibility, ELPs are generally well-tolerated and biodegradable. Their degradation occurs gradually in biological environments through both enzymatic and non-enzymatic mechanisms, including hydrolysis of labile bonds and redox-mediated cleavage [70], leading to the formation of low-molecular-weight products that can be metabolized and assimilated.

From an application perspective, their modular and tunable nature allows ELPs to be engineered to respond to environmental stimuli such as temperature and pH, or to self-assemble into nanoscale systems. From a translational standpoint, ELPs are inherently recombinant systems that enable precise control over sequence definition, molecular weight, and physicochemical properties. These features support their use in drug delivery, tissue engineering, and cancer therapy, while their biocompatibility minimizes the likelihood of systemic immune responses [71].

Thus, the choice of hydrophobic or hydrophilic amino acids as guest residue within the ELP sequence can influence the degradation and pharmacokinetic profile of the resulting nanosystem, including bioavailability or half-life in the blood stream. Hydrophobic residues are associated with slower degradation and lower renal filtration. In contrast, ELP sequences rich in polar amino acids (Lys, Arg) may enhance interactions with different tissues, thereby increasing the bioavailability of the ELP-based nanosystem. This tunability supports reproducibility and scalable production [72]. However, despite these strengths, the translation of ELP-based systems remains constrained by challenges related to purification processes, large-scale manufacturing, and regulatory considerations associated with recombinant biomaterials. In addition, further studies are required to comprehensively characterize their in vivo behavior, long-term safety, and degradation profiles under clinically relevant conditions. Current evidence does not report clinical trial data in humans for ELP-based drug delivery or cancer therapy; instead, research positions ELPs as promising candidates for future therapeutic development [73]. Recent work has also emphasized the importance of designing ELPs with improved stability and controlled pharmacokinetics to enhance their translational potential [74].

Preclinical evidence further indicates that systemically administrated ELP–drug nanoparticles preferentially localize at tumor sites through the permeability and retention effect, resulting in reduced toxicity and enhanced therapeutic efficacy [75].

In support of their preclinical translational potential, a genetically engineered ELP platform incorporating multiple copies of the IL-4 receptor-targeting peptide AP1 and the pro-apoptotic peptide KLAKLAK, termed AP1-ELP-KLAK, significantly inhibited tumor growth in several tumor xenograft models after systemic administration by inducing apoptosis, without specific organ toxicity; the same system also improved drug bioavailability, stability, membrane penetration, and drug half-life [75]. These preclinical findings suggest a favorable tolerability profile, although long-term immunogenicity and safety still require systematic clinical evaluation. Another preclinical approach using a bladder tumor-targeting peptide embedded in ELP as a drug delivery vehicle showed excellent localization in bladder tumor-xenografted mice after intravenous injection and remained confined to antigen-overexpressing tumor tissue [76].

In this regard, ELPs display temperature-responsive behavior: they are soluble at physiological temperatures but become insoluble at higher temperatures by coacervation, enabling hyperthermia-triggered nanoparticle assembly or injectable depot formation [77]. ELP block copolymers can be designed to assemble into nanoparticles in response to hyperthermia. In parallel, ELPs conjugated with radiotherapeutics can be injected directly into tumors, where they undergo coacervation to form intratumoral drug depots; these injectable ELP–radionuclide depots showed prolonged tumor residence and inhibition of tumor growth in preclinical studies [73,78]. Overall, while ELPs offer distinct advantages due to their recombinant origin and design flexibility, further efforts in process standardization and comprehensive biological evaluation are necessary to support their clinical implementation.

### 2.4. Ferritin: Biological Properties and Safety Considerations

Ferritin is a protein with a hollow, cage-like structure that plays a central role in iron storage and detoxification. It exhibits nanoscale dimensions, well-defined architecture, intrinsic biocompatibility, and an ability to encapsulate a wide variety of cargos. In addition, it shows high thermal and pH stability, as well as a strong capacity for functionalization, making it an attractive nanocarrier for bioactive molecules [79,80].

The endogenous origin of ferritin and its conserved biological function support its potential for clinical development [81]. However, several challenges remain for its translation. These include its natural biodistribution and preferential accumulation in organs such as the liver and spleen, which may result in off-target effects, as well as the potential induction of pro-inflammatory responses under certain pathological conditions, including cardiovascular and tumor-related diseases [82]. In addition, large-scale production, structural modification, and consistent functionalization of ferritin-based systems require further optimization.

Recent studies have also highlighted the need to better control receptor-mediated uptake and biodistribution in pathological environments, particularly in tumor settings [51,83]. In this context, ferritin systems that rely on CD71 for cellular internalization may generate off-target effects, since CD71 is not only overexpressed in malignant cells but also physiologically abundant in proliferating tissues such as bone marrow, intestinal epithelium, hepatocytes and activated immune cells, which can lead to unintended nanoparticle accumulation and reduced targeting specificity. Moreover, the presence of ferritin receptors in neuronal and endothelial cells at the blood–brain barrier suggests that exogenous ferritin-based nanocarriers could interact with sensitive neural compartments, raising additional safety considerations that should be incorporated into preclinical evaluations [84]. Ferritin-based nanoparticles have been widely investigated as drug delivery systems because of their biocompatible nanocage architecture, hollow interior, and ability to encapsulate therapeutic agents; however, their clinical advancement remains limited, and available clinical data are scarce and primarily focus on ferritin as a vaccine scaffold rather than as a drug delivery system [69]. Although human experimental data on ferritin-based systems exist, they remain limited and derive mainly from phase 1 clinical trials, including NCT04784767, NCT03186781, NCT04579250, and NCT03814720; importantly, these trials primarily evaluate ferritin as a vaccine scaffold, rather than as a drug delivery vehicle [69]. For instance, interactions mediated by receptors such as CD71, which is overexpressed in tumors but also present in normal proliferating tissues, may compromise selectivity [67]. Human H-ferritin exploits binding to transferrin receptor 1, CD71, for targeted delivery and imaging, and CD71 is described as overexpressed in metabolically active, iron-demanding cells, including tumor cells; however, high CD71 expression is also found in normal tissues with high metabolic activity and iron demand, such as liver, spleen, bone marrow, and the reticuloendothelial system [69]. This receptor distribution is consistent with biodistribution concerns: human H-ferritin showed a blood clearance time of only a few minutes in murine imaging studies and was primarily taken up by the reticuloendothelial system and lungs, with the highest concentrations at two hours observed in liver, spleen, and lungs [69].

Preclinical cancer studies nevertheless support the therapeutic potential of ferritin-based systems. In one ferritin-based nanocomposite hydrogel study, doxorubicin-loaded human ferritin, Dox@HFn, and oxidized dextran were used to form an injectable pH-sensitive hydrogel. After peritumoral injection, the hydrogel was retained locally for up to three weeks and gradually released intact Dox@HFn, facilitating tumor penetration through active transcytosis and inducing immunogenic cell death. When combined with anti-PD-1 antibody, this Dox@HFn hydrogel induced regression of orthotopic 4T1 breast tumors and generated systemic anti-tumor immunity capable of suppressing tumor recurrence and lung metastasis after surgical resection. The same study reported that combination with anti-CD47 antibody inhibited postsurgical recurrence in an aggressive orthotopic glioblastoma model and significantly extended mouse survival [85].

This point is reinforced by preclinical observations that ferritin can cross the blood–brain barrier via transcytosis in brain endothelial cells, supporting the need to assess CNS exposure and neurological safety when ferritin systems are administered systemically [85]. Clinically, the uploaded articles do not report a human trial of ferritin nanoparticles as a drug delivery platform. The available clinical evidence involving ferritin relates instead to serum ferritin as an iron-burden biomarker. In the BELIEVE phase 3 trial, registered as NCT02604433 and EudraCT 2015-003224-31, Luspatercept reduced transfusion burden in transfusion-dependent β-thalassemia, and the least-squares mean difference in serum ferritin at week 48 favored luspatercept by 348 µg/L [86]. This evidence relates to ferritin as a biomarker of iron metabolism and should not be interpreted as validation of ferritin-based nanocarriers. Overall, while ferritin presents favorable structural and biological features, further efforts are needed to address selectivity, safety, biodistribution, immune response, neurological implications, and manufacturability to support its clinical translation.

### 2.5. Apoferritin: Nanocarrier Properties and Design Considerations

Apoferritin is the iron-free form of ferritin, consisting of a 24-subunit protein nanocage that self-assembles into a spherical structure approximately 12 nm in diameter. Its internal cavity (~8 nm) and negatively charged interior enables efficient encapsulation of small molecules, peptides, and nanoparticles, while maintaining structural stability under a wide range of pH and temperature conditions. Functionally, apoferritin combines high biocompatibility [87], low inherent-toxicity [88,89] and biodegradability as it is processed through physiological protein degradation pathways. Its cellular uptake is primarily mediated via transferrin receptors (TfR1) [88], which are frequently overexpressed in tumor cells, supporting its use in targeted delivery strategies. In addition, its reversible pH-dependent disassembly and reassembly facilitates drug loading and controlled intracellular release [90]. However, despite these advantages, several limitations remain. Dependence on TfR1-mediated uptake may reduce specific accumulation due to receptor expression in non-tumor proliferating tissues. Furthermore, control over cargo loading efficiency and release kinetics can vary depending on formulation conditions, potentially affecting reproducibility. In apoferritin-based systems, it is crucial to consider both encapsulation efficiency and the risk of premature cargo leakage. Since encapsulation relies on pH-induced dissociation and reassembly, the final yield can vary depending on experimental conditions and drug properties [90]. Furthermore, although apoferritin provides a stable cavity for retaining therapeutic molecules, stability studies have shown that premature release is not entirely eliminated: for example, a study of ApoDox formulations determined up to maximum of 7.5% cargo leakage, equivalent to a retention efficiency of 92.5% during storage or circulation [91]. Similarly, in mineralized or coated formulations, instability of the inorganic material can promote partial release into the bloodstream, compromising therapeutic efficacy. These limitations underscore the need to optimize loading conditions and the structural stability of apoferritin to minimize unwanted release in vivo.

Another result shows that apoferritin exhibits moderate encapsulation efficiencies, typically between 50% and 71%, depending on the incorporated inhibitor (e.g., Neq0551 with 55.2%, Neq0554 with 50.9%, and Neq0568 with 71.2%). These values reflect the limited nature of the passive diffusion process through the protein’s hydrophobic channels, implying that a significant fraction of the added drug is not retained within the cavity. Furthermore, the study demonstrates that cargo release is highly pH-dependent, with accelerated release under acidic conditions: for Neq0554, over 50% of the compound is released within the first 6 h at pH 5.5 and over 75% after 12 h, while release is much lower at physiological pH (∼32%) [92]. These data highlight limitations in loading efficiency and drug stability.

From a translational perspective, apoferritin benefits from its endogenous origin and well-characterized structure, but challenges persist in large-scale production, standardization of loading protocols, and consistent functionalization. Overall, apoferritin represents a promising nanocarrier platform, particularly for targeted delivery applications, although further optimization is required to improve specificity and clinical feasibility.

### 2.6. Transferrin: Biological Properties and Safety Considerations

Transferrin (Tf) is an approximately 80 kDa glycoprotein involved in iron transport and homeostasis. It circulates in the bloodstream, preventing iron precipitation and limiting the formation of reactive oxygen species. An additional limitation of CD71-targeting strategies is direct competition with endogenous transferrin, the receptor’s physiological ligand. Under normal conditions, transferrin circulates at high concentrations of 30–300 ng·mL^−1^ (67.4–674.1 pmol·L^−1^) [93] and dynamically occupies this receptor’s binding sites, which can reduce the receptor’s availability for exogenous nanocapsids such as apoferritin. This competition can decrease the efficiency of tumor targeting, favor uptake in healthy tissues with high iron demand, and limit the selective internalization of the nanocarrier in cancer cells. Therefore, the design of CD71-based systems must consider the receptor’s basal saturation and the need to optimize affinity, binding kinetics, and mechanisms for evading circulating transferrin. Iron delivery to cells occurs via transferrin receptor 1 (TfR1)-mediated endocytosis [79]. TfR1 expression is often upregulated in tumor cells and has been associated with increased malignancy, reflecting the elevated iron demand required for cell proliferation [94]. While this feature supports the use of transferrin in targeted delivery systems, it also introduces important limitations, particularly in the context of exogenous administration, as TfR1 is not tumor-specific and is expressed in various normal proliferating tissues. Indeed, TfRs are reported in normal erythrocytes, hepatocytes, intestinal cells, epithelial cells of the choroid plexus, neurons and the BBB, whereas cancer cells may display markedly higher expression, in some cases up to 100-fold above normal cells [95]. In pancreatic cancer, TfR has also been described as a type II transmembrane glycoprotein expressed by malignant cells in approximately 90% of cases, supporting its relevance as an active-targeting receptor in this tumor type [96]. In addition, TfR1 is involved in signaling pathways related to inflammation, cell growth, and immune regulation, which may further influence the biological response to transferrin-based systems. The degradation of transferrin-based nanoparticles involves both the breakdown of the carrier system and the recycling of transferrin through endosomal pathways, leading to the release of iron and therapeutic cargo. Hybrid systems, such as polymer- or lipid-based nanoparticles incorporating transferrin, generally exhibit favorable biocompatibility and stability over time [97]. Preclinical studies support the feasibility of this approach: T7/TfR-targeted siRNA nanoparticles and micelles have shown improved BBB permeability, enhanced cellular uptake, increased accumulation in brain tumors and superior therapeutic activity compared with non-targeted systems in glioma models [98]. More recent preclinical work in glioma reported T7-C-coated micelles loaded with slit2 siRNA, tested in GL261, BV2 and 293T cells and in GL261 glioma-bearing C57BL/6 mice, where intranasal delivery mediated by TfR binding inhibited glioma tumor growth. Another T7-C/siRNA complex was reported to target TfR on glioma cells and deliver slit2 siRNA through the olfactory bulb pathway, converting the tumor microenvironment from “cold” to “hot” after delivery [99]. However, the available review literature indicates that clinical translation remains limited for Tf/TfR-targeted nanocarriers: despite numerous publications, only a few viable products have entered clinical trials, and further preclinical and clinical research is still needed to define optimal nanocarrier design, ligand density, size, surface properties and BBB-crossing efficiency [98].

### 2.7. Encapsulin: Biological Properties and Safety Considerations

Encapsulins are bacterial protein nanocompartments that self-assemble into highly stable spherical structures typically ranging from 20 to 45 nm [100]. Their native biological role involves encapsulating enzymatic cargo via specific peptide tags, a feature that can be harnessed for the incorporation of therapeutic proteins or functional molecules [100,101]. Encapsulins offer several advantages, including precise structural definition, genetic programmability, and ease of recombinant production, enabling scalable and reproducible fabrication. Their modular architecture allows for independent engineering of the internal cargo and external surface, supporting applications in targeted delivery, nanoreactors, and diagnostic systems profile [102,103]. Repeated administration of encapsulins should take into account their marked intrinsic immunogenicity, as these bacterial nanocompartments robustly activate the adaptive immune response. In a study [104] on p30-encapsulin, the nanoparticles induced strong dendritic cell maturation, a significant increase in follicular helper T cells and germinal center B cells, and the generation of high-affinity antibodies capable of persisting in lymph nodes. Consistently, subsequent work with p54-encapsulin showed even more potent humoral and cellular responses than the monomeric antigen, including marked increases in IgG1 and IgG2a, sustained DC activation, and expansion of Tfh and GCBs, along with prolonged antigen retention in germinal centers [105]. Although this immunogenicity is advantageous in the vaccine context, in therapeutic applications or with repeated administration it could promote the development of anti-nanoparticle antibodies, accelerate systemic clearance, reduce nanocarrier efficacy, and increase the risk of secondary inflammatory reactions. Therefore, the continued use of encapsulins as a delivery platform requires strategies that modulate or attenuate these cumulative immune responses.

This immunogenicity may limit repeated administration [103]. In addition, their in vivo behavior, biodistribution, and long-term safety profiles remain insufficiently characterized, which limits their immediate translational potential, as current studies highlight incomplete understanding of bio–nano interactions and clearance mechanisms [106]. From a translational standpoint, encapsulins benefit from straightforward recombinant production and high structural uniformity, enabling scalable and reproducible fabrication [106]. However, regulatory challenges associated with non-human protein systems and the need for extensive safety validation represent critical barriers, particularly given the limited clinical data and early-stage development of these platforms [107].

From a translational standpoint, the available evidence remains predominantly preclinical, with no human clinical trial data identified in the reviewed articles. In a systemic in vivo study, purified Thermotoga maritima encapsulin nanocages (TmEnc) were intravenously administered to healthy BALB/c mice and showed colloidal stability, storability, and blood compatibility. The formulation displayed an encouraging nanosafety profile, with no abnormal weight loss or gross pathology, although temporary alterations in toxicity biomarkers were observed. Importantly, TmEnc induced nanocage-specific IgM and IgG antibodies, confirming immunogenicity, but without prolonged pro-inflammatory effects. Biodistribution analysis showed sequestration from blood circulation by the liver, followed by biodegradation within Kupffer cells, indicating clearance through the mononuclear phagocyte system [103]. In a separate preclinical oncotherapy study, a genetically encoded two-step delivery system using doxorubicin-loaded T. maritima encapsulin nanoparticles targeted HER2-overexpressing tumors through affibody-mediated pre-targeting and SpyTag/SpyCatcher ligation. The system demonstrated high specificity, with approximately an order-of-magnitude greater binding to HER2-positive cells than control cells, and in vivo studies reported 95% tumor growth inhibition, compared with 62.3% for one-step delivery and 87.5% for free doxorubicin. The study also reported elimination of doxorubicin-associated heart toxicity while maintaining anticancer activity [49]. These findings support the therapeutic potential of encapsulin-based platforms but also reinforce that translation remains at an early stage, requiring further pharmacokinetic, repeated-dose immunogenicity, biodistribution, clearance, and long-term safety studies before clinical application.

### 2.8. VLPs: Biological Properties and Safety Considerations

Virus-like particles (VLPs) are nanoscale protein assemblies derived from viral capsid proteins that retain the structural organization of viruses but lack genetic material, rendering them non-infectious [108]. Their size typically ranges from 20 to 200 nm [109], and their highly ordered architecture allows both surface functionalization and internal cargo loading. A key advantage of VLPs is their strong immunogenicity, which enables efficient activation of the immune system and supports their widespread use in vaccine development [110]. In the context of nanomedicine, this property can be exploited for cancer immunotherapy and targeted delivery applications. Additionally, VLPs are biodegradable and can be produced with high structural uniformity [111]. However, their intrinsic immunogenicity also represents a limitation, particularly for drug delivery applications where immune activation may lead to rapid clearance or adverse reactions [108]. Furthermore, pre-existing immunity against viral components and batch-to-batch variability in production may complicate their clinical use, especially in the context of scalable manufacturing and reproducibility [112,113]. Nevertheless, extending their application to drug delivery and oncology requires careful modulation of their immunological properties and optimization of manufacturing processes [114].

Despite these limitations, from a translational perspective, VLPs represent one of the most clinically advanced protein-based nanoplatforms, particularly in prophylactic vaccination, with approved vaccines against HBV and HPV and growing investigation in cancer immunotherapy [115]. This clinical maturity is supported by recent HPV vaccine trials. In the PRIMAVERA immunobridging trial (NCT03728881), a single dose of AS04-adjuvanted bivalent HPV vaccine in girls aged 9–14 years was compared with three doses of quadrivalent HPV vaccine in women aged 18–25 years. Seropositivity remained close to 100% at 36 months, but non-inferiority was demonstrated for HPV-18 and not for HPV-16; serious adverse events were considered unrelated to vaccination [116]. Similarly, a phase III randomized trial of an *Escherichia coli*-produced bivalent HPV16/18 vaccine (NCT01735006) reported 100.0% efficacy against HPV16/18-associated high-grade genital lesions and 97.3% efficacy against persistent infection after 66 months, with no serious adverse events considered vaccine-related [117]. These data confirm the clinical safety and efficacy of VLP-based prophylactic vaccines. However, therapeutic cancer vaccines and drug delivery applications remain less mature, with most oncology-focused VLP evidence still preclinical or early-stage [115]. For instance, polyionic papillomavirus VLP vaccines induced robust CD8+ T-cell responses in peripheral blood and tumor tissues and showed efficacy in a murine prostate cancer model [118]. Therefore, although VLPs are clinically validated as vaccine platforms, their broader use in cancer immunotherapy and targeted delivery requires further optimization of immunogenicity, biodistribution, manufacturing reproducibility, and long-term safety.

There are multiple biological systems used to express and self-assemble VLPs, which markedly dictate their yield, structural fidelity, post-translational modifications and eventual clinical safety profile [109]. The most typical expression systems range from bacteria (*E. coli*), plants (Tobacco Mosaic Virus), insect cells (Baculovirus) and mammalian cells (lentivirus) [111]. To effectively translate a VLP into a targeted cancer therapeutic, the protein shell must be modified to carry tumor antigens or payloads [111]. Thus, genetic fusion (Chimeric VLPs) of small tumor antigens spliced directly into the viral capsid genes, chemical/bioorthogonal conjugation of neoantigens or targeting antibodies to be displayed on the surface, and internal cargo encapsulation are the gold-standard strategies for functional modification of VLPs [112]. Overall, VLPs offer significant potential, particularly in immunotherapy, but require context-specific design strategies to balance efficacy and safety. Thus, VLPs are suitable nanosystems for mechanistic applications in oncology, such as therapeutic cancer vaccines, targeted drug delivery, and in situ tumor microenvironment (TME) modulation.

### 2.9. Emerging Protein-Based Nanocarriers: Biological Properties and Safety Considerations

Hybrid systems combining proteins with synthetic polymers (e.g., PEGylated proteins or protein–polymer conjugates) represent a key translational strategy to improve stability, circulation time, and drug loading capacity [119,120]. Among these, polyethylene glycol (PEG) is one of the most widely used polymers due to its high biocompatibility, solubility in aqueous and organic media, low toxicity, and reduced immunogenicity [121,122].

Despite these advantages, PEGylation presents important limitations. Notably, the lack of metabolic degradation pathways may lead to prolonged retention of PEGylated conjugates, resulting in accumulation in tissues and clearance through hepatobiliary routes [120]. Additionally, immune responses against PEG and variability in biological performance have raised concerns regarding its long-term safety and clinical reliability. Thus, the formation of anti-PEG antibodies represents a major hurdle in the development of protein–polymer conjugates, since it dismantles the historical paradigm that PEG is a completely inert, stealth, and non-immunogenic material. Consequently, alternative strategies to PEGylation are being actively explored. These include structural PEG analogues such as poly(oligo(ethylene glycol)methacrylate), hydrophilic polymers such as poly(N-(2-hydroxypropyl)methacrylamide) and poly(N,N-dimethylacrylamide), as well as naturally derived polymers such as polysialic acid and hyaluronic acid, and recombinant polypeptide-based systems such as PASylation [122].

These advanced conjugation strategies enable improved control over pharmacokinetics while preserving biological functionality and targeting capabilities [123]. However, despite their potential, protein–polymer conjugates still face significant challenges related to structural heterogeneity, batch-to-batch reproducibility, and regulatory classification, primarily due to the inherent complexity of hybrid biomolecular systems [120].

Another emerging strategy to enhance immune evasion and biological targeting is based on membrane-coated nanocarriers incorporating protein components [124,125]. By mimicking cellular membranes, these systems can improve circulation time, reduce immune recognition, and promote homotypic targeting [125,126]. However, their structural and compositional complexity introduces challenges in large-scale manufacturing, quality control, and standardization, which currently limit their clinical translation [124,127]. There are also other types of biomaterials used in protein nanoparticle engineering, incorporating other relevant families that have demonstrated utility in nanomedicine. Among these are zeins, hydrophobic proteins derived from maize that can self-assemble into biodegradable nanoparticles with high stability (Liu et al., 2023) [128]; antimicrobial peptides, whose use arose as a response to the problem of antibiotic resistance [129], which possess broad-spectrum activity against multiple types of microorganisms and can also be combined with other biological polymers, such as elastin-like, which promote self-assembly and provide inherent biological functionality to the resulting nanostructures [130]; gelatin, a partially hydrolyzed collagen widely used for its biocompatibility, cross-linking capacity, and chemical versatility and approved by the FDA as a safe pharmaceutical excipient [131]; and mucins, a highly hydrated proteoglycan that allows for modulation of interactions with biological barriers presents in epithelia including the corneal surface, the gastrointestinal tract, and the female reproductive system [132], and are being explored as emerging platforms for develop hydrogels and nano carriers. These examples provide a more complete view of the diversity of natural proteins used in PNP engineering and biomedical applications.

Taken together, natural and recombinant protein-based nanomaterials present distinct translational trade-offs, where naturally derived systems offer established biocompatibility and clinical familiarity, while recombinant platforms provide enhanced control, reproducibility, and design flexibility. This comparison highlights the importance of considering not only material properties but also manufacturability, biological behavior, and clinical feasibility when evaluating their potential for translation. These biological and safety characteristics directly influence the design and performance of protein-based systems in advanced applications, particularly in theranostics and drug delivery.

## 3. Protein-Based Nanomaterials for Theranostics

Theranostic nanoparticles (NPs) are multifunctional nanosystems that integrate diagnostic and therapeutic capabilities within a single platform, enabling simultaneous disease detection, treatment, and monitoring of therapeutic response [50]. For translational relevance, such systems must satisfy certain criteria, including biocompatibility, biodegradability, selective accumulation at the pathological site, and predictable clearance into non-toxic byproducts [133,134]. Within this framework, theranostics represents a precision-medicine approach integrating diagnostic imaging and targeted therapy to enable patient stratification, treatment selection, and longitudinal response monitoring [135]. Theranostic systems are best described by combining platform-based classification (e.g., radiotheranostic and nanotheranostic systems) with their underlying delivery mechanisms, including passive and active targeting as well as stimuli-responsive activation, all of which jointly govern their in vivo behavior and therapeutic performance [136]. Its most established form is radiotheranostics, in which a targeting vector such as a peptide, antibody, or small molecule is coupled to diagnostic or therapeutic radionuclides to allow for selective visualization and treatment of diseased tissue. Within this context, theranostic approaches are generally categorized into radiotheranostic, nanotheranostic, and other molecularly targeted platforms, with radiotheranostics representing the most clinically advanced modality. Therapeutic radionuclides exert their effects primarily through ionizing-radiation-induced cytotoxicity, including DNA damage, with the biological outcome depending on the emitted particle type and linear energy transfer. In contrast, nanotheranostic platforms employ multifunctional nanocarriers, such as liposomes, dendrimers, or metallic nanoparticles, to co-deliver imaging probes and therapeutic agents, often incorporating stimuli-responsive release and radiosensitizing functionalities [137]. Beyond stimuli-responsive behaviour, nanotheranostic delivery also relies on passive and active targeting mechanisms. Passive targeting is mainly mediated by the enhanced permeability and retention effect, whereas active targeting depends on ligand–receptor interactions that promote selective uptake by diseased cells [138]. In practice, many nanotheranostic systems integrate passive accumulation, receptor-mediated internalization, and stimulus-triggered payload release to improve tumor localization, intracellular delivery, and therapeutic response [139]. Mechanistically, theranostic agents proceed through target recognition, systemic distribution, selective accumulation, cellular internalization, and therapeutic activation, while simultaneously allowing for real-time monitoring of treatment response. This dual-function approach supports more individualized therapy and may reduce off-target toxicity in oncology and other disease areas [140].

To enable a direct comparison across different platforms, the following sections evaluate representative protein-based nanomaterials according to a common set of criteria, including imaging capability, therapeutic integration, targeting strategy, and translational feasibility.

A wide variety of theranostic nanosystems have been proposed, including protein-based carriers [141], liposomes [142], inorganic nanoparticles [143], polymeric systems [144], and hybrid platforms [145]. In this context, this review focuses on the most representative protein-based nanomaterials for theranostics: albumin, silk fibroin, elastin-like polypeptides, ferritin, and transferrin, along with VLPs, protein–polymer hybrids, and membrane-coated nanocarriers. These platforms offer significant advantages, such as biocompatibility, functionalization capacity, molecular recognition, and the integration of diagnostic and therapeutic functions. However, from a regulatory standpoint, their development remains particularly challenging, as they combine the complexity of biologic drugs with the uncertainties inherent to nanomaterials. The main challenges include product classification, characterization of critical attributes, immunogenicity, biodistribution, manufacturing reproducibility, demonstration of comparability, and clinical validation of the theranostic function.

Therefore, progress toward clinically viable platforms will depend on an integrated development strategy based on scalable designs, nanospecific quality controls, translational preclinical studies, and clinical evidence demonstrating both therapeutic safety and diagnostic utility.

**Figure 1:**
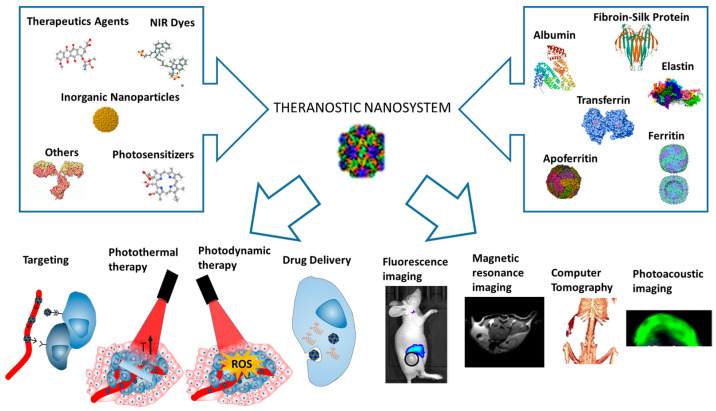
Schematic representation of protein nanoparticles for imaging and cancer therapy based on multimodal photothermal therapy. Adapted with permission from Sun et al. [146,147].

Accordingly, rather than providing an exhaustive list of examples, this review identifies design strategies, implementation challenges, and comparative performance criteria to guide the development of protein-based theranostic platforms toward clinically viable solutions. Representative approaches are summarized in Figure 1 [146,147].

### 3.1. Albumin

Albumin is the most abundant serum protein and functions physiologically as a natural carrier for numerous endogenous and exogenous molecules. Its high biocompatibility, long circulation half-life, and amphiphilic character make it an ideal scaffold for theranostic nanoplatforms. Albumin can also enhance tumor accumulation through gp60-mediated transcytosis and interaction with SPARC proteins in the tumor microenvironment [148].

Bovine serum albumin (BSA) is widely used as an experimental model due to its availability and ease of functionalization. Beyond passive drug delivery, BSA acts as a nanoreactor, enabling the in situ formation of inorganic nanostructures, as Gd_2_O_3_ and CuS [149]. Hybrid BSA-based systems incorporating gadolinium, copper sulfide, or manganese oxide combine MRI, photoacoustic imaging, and photothermal therapy (PTT) [150].

More advanced albumin-based platforms incorporate photosensitizers such as chlorin e6 by carbodiimide coupling. These systems enable synergistic photodynamic and photothermal therapy (PDT/PTT), showing a potent hyperthermia and remarkable photo-induced damage and intracellular ROS against the tumors being irradiated, with photodynamic and photothermal damage resulting in an anticancer efficacy in 4T1 murine breast cancer cells [151]. A combination of strategies was used to combine a (Gd_2_O_3_/CuS NDs) core–shell structure with BSA, to which Cy7.5, a near-infrared fluorescent dye, was added via conjugation with the amino groups of albumin, creating a multifunctional system capable of generating near-infrared fluorescence images, magnetic resonance imaging (MRI), and simultaneous photothermal tumor ablation. This platform achieved complete tumor elimination without recurrence in mice bearing 4T1 tumors [152]. More recently, Ce_6_-conjugated BSA was used to generate Ce_6_IrO_2_/MnO_2_ core BSA nanoparticles via a biomineralization process. Due to the presence of IrO_2_, this system exhibits high photothermal conversion efficiency and is suitable for computed tomography (CT) imaging [153]. Targeting ligands, such as folic acid, can be incorporated to enhance tumor uptake [154,155]. Using this, folic acid-modified BSA was employed to create a hybrid platform with MnO_2_. Highly fluorescent carbon quantum dots (CQDs) and doxorubicin (Dox) were then added, forming a nanosystem for magnetic resonance imaging and photoluminescence, as well as for inhibiting cancer migration. This system showed a better therapeutic effect against cancer compared to pure anticancer drugs under the same conditions, exhibiting antimigratory properties in both in vitro and in vivo experiments with HeLa cells [156]. A BSA matrix was also used to improve the stability of lysozyme-mediated agglomerates of iron oxide core–gold shell nanoparticles and provide an environment suitable for the encapsulation of Dox, thus obtaining a platform with appreciable blood serum stability. The presence of BSA also helped to efficiently load and deliver the chemotherapeutic drug to cancer cells, exhibiting a significant killing effect. The resulting nanocarrier combines plasmonic photothermal therapy and anticancer drug delivery with a magnetic targeting and luminescence-based imaging capability, and has exhibited in vitro theranostic capabilities in HeLa cells [157]. However, receptor heterogeneity and saturation effects may limit the robustness of active targeting strategies in clinical settings.

It is important to note that albumin-based platforms also enable therapeutically relevant combination strategies. Albumin-stabilized gold nanoclusters co-loaded with doxorubicin and SN38 provide dual stimulus-responsive release and effectively reduce cancer stem cell populations, as demonstrated by decreased mammosphere formation. This highlights the potential of albumin nanocarriers to address tumor relapse and drug resistance, reinforcing their status as one of the most well-established platforms [158].

While these systems demonstrate strong multimodal performance, their increasing compositional complexity raises concerns regarding reproducibility and scalability, and may pose additional challenges in terms of regulatory approval.

From a translational perspective, human serum albumin (HSA) offers clear advantages, including reduced immunogenicity and well-established clinical use. HSA-based nanoplatforms have enabled NIR-II imaging-guided therapy [159], synthesizing Ag_2_S nanodots inside HSA nanocages, MRI-guided treatments [160,161], and there are several examples of theranostic nanosystems synthesized following this method, with HSA being used as a natural carrier for photosensitizers [162,163,164,165]. More recently, HSA-phthaloNO_2_ nanoparticles were formed through a desolvation process, with HSA being selected for its biodegradable and nontoxic properties. To achieve specific targeting, these HSA nanoparticles were biofunctionalized with anti-FRα antibodies, since folate receptor alpha (FRα) is overexpressed at the surfaces of nearly all ovarian cancer cell lines. The result was a phototheranostic agent for fluorescence imaging and photothermal and photodynamic therapy (PDT) of ovarian cancer, demonstrating high efficiency in killing A2780 cancer cells and showing anticancer activity in in vivo xenograft tumors of the human ovarian adenocarcinoma cell line NIH:OVCAR-3 [166].

Beyond small-molecule ligands, antibody-functionalized albumin-based nanoparticles have also been explored to enhance targeting specificity and imaging contrast. However, while such strategies can yield promising preclinical outcomes, they substantially increase system complexity, which may ultimately limit scalability and clinical translation when compared with simpler albumin-based designs.

BSA is predominantly employed as an experimental model owing to its wide availability, cost-effectiveness, and high tolerance to chemical modification, which make it particularly suitable for proof-of-concept studies, mechanistic investigations, and optimization of multifunctional nanoreactors. In contrast, HSA offers clear advantages for clinical translation, including minimal immunogenicity, well-established pharmacokinetics, and regulatory familiarity arising from its extensive use in approved formulations. Accordingly, HSA-based theranostic systems frequently rely on simpler architectures based on self-assembly or non-covalent complexation, can enhance scalability and improved batch-to-batch reproducibility while retaining effective imaging-guided therapeutic performance, as has been particularly demonstrated in NIR-responsive and MRI-guided modalities [158,159,160,161,163,164]. This distinction highlights a recurrent trend in albumin-based theranostics, where BSA-derived systems drive innovation and functional complexity at the preclinical stage, whereas HSA-based platforms represent the most realistic pathway toward clinical implementation.

Indeed, the clinical trajectory of albumin exemplifies this benchmark most concretely, spanning FDA-approved therapeutics, diagnostic imaging agents, and next-generation multimodal constructs. Human serum albumin (HSA) has emerged as one of the most clinically validated scaffolds for the development of oncological drug delivery and theranostic systems, owing to its biocompatibility, long circulatory half-life mediated by the neonatal Fc receptor (FcRn), and intrinsic tumour-targeting properties through the gp60/caveolin-1/SPARC transcytosis pathway [167]. The most paradigmatic example of clinical translation is nab-paclitaxel (Abraxane^®^), an albumin-stabilised nanoparticle formulation of paclitaxel approved by the U.S. Food and Drug Administration (FDA) for the treatment of metastatic breast cancer, locally advanced or metastatic non-small cell lung carcinoma (NSCLC), and metastatic adenocarcinoma of the pancreas [167,168]. The therapeutic advantage of Abraxane^®^ relies on receptor-mediated transcytosis: albumin binds to the gp60 receptor on the intraluminal endothelial cell membrane, activating caveolin-1 and initiating the formation of caveolae that transport the albumin–drug complex into the tumour interstitium, where the secreted protein acidic and rich in cysteine (SPARC) retains albumin and releases the cytotoxic payload [167]. Beyond chemotherapy, albumin-based platforms have been clinically validated in diagnostic imaging: Optison^®^ (GE Healthcare, Chicago, IL, USA) is an FDA-approved ultrasound contrast agent composed of human albumin microspheres encapsulating perfluoropropane, representing one of the earliest approved applications of a protein-shell microbubble in clinical diagnostics, while Nanocoll^®^ constitutes a radiolabelled albumin colloid employed in SPECT-based sentinel node lymphoscintigraphy [169]. Advancing towards a combined theranostic concept, aldoxorubicin (LADR-x; LadRx Corporation, Los Angeles, CA, USA), an albumin-binding doxorubicin prodrug designed to exploit circulating endogenous albumin for tumour-selective drug delivery, is currently under regulatory review for FDA marketing authorisation in soft-tissue sarcomas [169].

This balance between functional versatility and clinical feasibility provides a useful benchmark for evaluating other protein-based nanomaterials discussed in the following sections, such as silk fibroin, which offer distinct material advantages but face different translational limitations.

### 3.2. Silk

In contrast to albumin based nanoplatforms, which currently represent the most clinically mature protein carriers for theranostic applications, silk fibroin (SF) has emerged as a highly versatile protein–polymer primarily driven by its outstanding mechanical properties, biodegradability, and tunable physicochemical behavior. This versatility enables the fabrication of nanoparticles, nanofibers, and hybrid systems capable of integrating imaging agents and therapeutic cargos, positioning silk fibroin as an attractive platform for advanced preclinical theranostic design rather than near term clinical translation [170].

SF-based nanocarriers have demonstrated potential in fluorescence- and MRI-guided photothermal and photodynamic therapies. For example, indocyanine green (ICG) has been loaded into SF nanoparticles via a desolvation process to produce ICG-dyed SF nanoparticles, which have been successfully applied in C6 glioma models [171]. In another approach, exploiting the electrostatic interaction between negatively charged SF (under neutral aqueous conditions) and polyethyleneimine (PEI)-coated gold nanoparticles (AuNPs), AuNPs/SF nanofibers were fabricated. These composites significantly enhanced the photothermal therapeutic efficiency of AuNPs and effectively inhibited tumor growth in an MCF-7 xenograft mouse model [172]. Biomineralization strategies have further expanded their multimodal functionality. For instance, MnO_2_ mineralization combined with the incorporation of ICG and doxorubicin (Dox) at active amino residues on SF has enabled the development of multifunctional theranostic platforms integrating photothermal therapy (PTT), photodynamic therapy (PDT), and chemotherapy, guided by fluorescence and MR imaging [173]. And more complex designs further integrate these therapies; a recent study reported that silk fibroin nanoparticles doped with MnO_2_ and functionalized with polyethylene glycol and folic acid exhibited enhanced stability and, consequently, improved antitumor activity. When co-loaded with Dox and chlorin e6 (Ce_2_) as chemotherapeutic and photosensitizing agents, respectively, these nanocarriers effectively inhibited the proliferation of MCF-7 cells under laser irradiation. Furthermore, in vivo experiments revealed preferential tumor accumulation, which significantly suppressed breast cancer progression [174].

Despite these promising attributes, the translational trajectory of silk fibroin nanoplatforms remains limited. Key challenges include source-dependent variability, batch-to-batch inconsistencies, difficulties in endotoxin control, and limited scalability under GMP conditions. Additionally, their non-human origin may introduce additional regulatory considerations. As a result, silk fibroin systems remain predominantly at an early preclinical stage [171,175].

### 3.3. Elastin-like Polypeptides

Elastin-like polypeptides (ELPs) are thermally responsive biopolymers derived from natural elastin. This unique property enables precise control over self-assembly and stimuli-responsive drug delivery. Nevertheless, despite this high degree of molecular adaptability, ELP-based systems are still relatively far from clinical application in the field of theranostics [176].

Theranostic systems based on ELP have been proposed by combining elastin-like polypeptides, using the gene sequence (VGVPG), with an N-terminal cysteine residue and gold nanoparticles, obtaining a nanostructure that allowed for multimodal imaging and photothermal therapy in C8161 tumor cells in a murine model after intratumoral injection [147]. Targeted platforms incorporating tumor-targeting peptides and chemotherapeutic agents have also been designed, enabling highly specific, image-guided combination therapies. One such example involves the F3 peptide, the N-terminal fragment of human high-mobility group nucleosomal binding protein 2 (HMGN2). F3 selectively binds to nucleolin, which is overexpressed on the surface of tumor cells and tumor-associated endothelial cells. Following binding, F3 is internalized and translocated to the nucleus, making it an effective targeting ligand.

Taking advantage of this mechanism, a chimeric elastin-like polypeptide (ELP) genetically incorporating the F3 fragment was engineered and subsequently loaded with polypyrrole (PPy) and doxorubicin (Dox). The resulting nanoparticles (Dox/PPy-ELP-F3) exhibited strong potential as a theranostic platform, enabling photoacoustic and thermal imaging-guided synergistic photothermal and chemotherapy. Notably, the photothermal effect generated by PPy could accelerate the release of Dox, enhancing therapeutic efficacy. In vivo studies in C8161 tumor-bearing mice demonstrated that, when combined with laser irradiation, Dox/PPy-ELP-F3 achieved highly efficient and specific melanoma treatment, with no observable side effects, highlighting its promise as a targeted theranostic system [177].

Despite their exceptional molecular precision and tunability, ELPs face challenges related to recombinant production, cost, and optimization of in vivo pharmacokinetics and stability. As a result, they remain in the early stages of translational development.

### 3.4. Ferritin

Halfway between established albumin platforms and earlier-stage protein materials such as silk fibroin or elastin-like polypeptides, ferritin is a naturally occurring protein nanocage composed of 24 subunits that offers a highly uniform, biocompatible, and functionally versatile scaffold for advanced theranostic applications [178,179].

Its ability to bind transferrin receptor 1 (Tfr1) allows for receptor-mediated cellular uptake [178], while its reversible assembly enables efficient drug loading.

Apoferritin (AFN) is a particularly versatile platform for cancer therapy. AFN is an iron-free form of ferritin that retains its characteristic hollow cage structure, suitable for the encapsulation of therapeutic and diagnostic agents. The inner surface of recombinant heavy-chain apoferritin (HFn) exhibits a high negative charge density [179], which facilitates cargo loading. Moreover, HFn specifically binds to transferrin receptor 1 (TfR1), a receptor overexpressed in many cancer cells, thus promoting receptor-mediated internalization via clathrin-coated pits [180].

Apoferritin-based nanoplatforms have been used for CT, MRI, and fluorescence imaging-guided therapies, including photothermal and chemotherapeutic approaches. More advanced designs incorporate catalytic or microenvironment-responsive elements to enhance therapeutic efficacy.

AFN can also be reversibly disassembled under extreme pH conditions (pH 2–3 or 10–12), enabling efficient cargo loading followed by reassembly into a stable nanocage. This property has been exploited to construct multifunctional nanostructures. For example, doxorubicin (Dox)-loaded AFN nanocages have been used as templates for the in situ growth of bismuth sulfide crystals, yielding nanoparticles capable of computed tomography (CT) imaging-guided chemo- and radiotherapy, which effectively induced HeLa cell death both in vitro and in vivo [181].

Further functionalization of Dox-loaded AFN with the near-infrared (NIR) fluorescent dye ADS-780 resulted in the formation of nanoparticles, which combined imaging and therapy and showed promising efficacy in HT-29 cells and murine models [182]. Similarly, AFN has been employed as a nanoreactor for the synthesis of CuS-based nanoparticles functionalized with NIR dyes, allowing for simultaneous fluorescence imaging and photothermal therapy (PTT), with the additional advantage of using distinct optical wavelengths for imaging and therapeutic activation. This system demonstrated efficacy in U87MG human glioma cells and in Ehrlich ascites carcinoma models [183].

**Figure 2:**
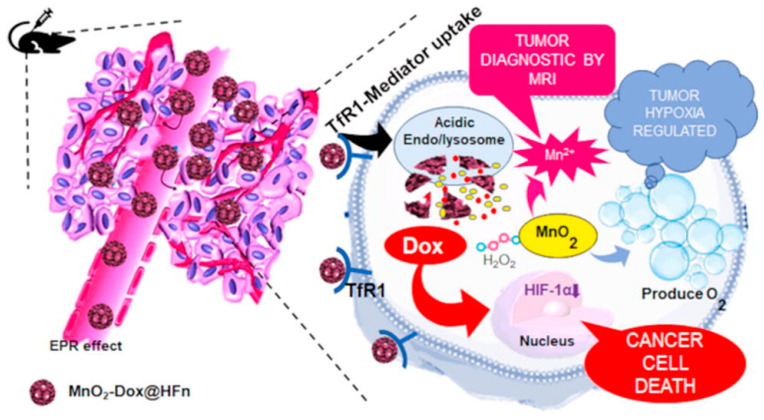
Schematic illustration of MnO_2_-Dox@HFn for tumor specific MR imaging-guided treatment and double anti-cancer activity due to the intracellular oxidase damage and chemotherapeutic effect of Dox. Reproduced with permission from Veroniaina et al. [182].

To address tumor hypoxia, MnO_2_ and Dox have been co-encapsulated into HFn nanocages to form MnO_2_-Dox@HFn via a self-assembly approach, generating nanozymes upon pH adjustment. These systems exhibit peroxidase- and catalase-like activities, enabling in situ O_2_ production and thereby alleviating hypoxia in the tumor microenvironment. As a result, enhanced therapeutic efficacy and tumor-specific magnetic resonance (MR) imaging guidance were achieved, with higher cytotoxicity in HeLa and 4T1 cells compared to free Dox and a tumor inhibition rate of up to 78.5% in vivo (Figure 2) [184].

Finally, targeting capabilities have been further improved by conjugating folic acid (FA) to the AFN surface, together with the NIR-II dye IR1061, followed by loading with paclitaxel (PTX). This multifunctional platform combines NIR-II-mediated PTT and chemotherapy while overcoming solubility limitations of the individual components. The system exhibits excellent aqueous stability and efficient cellular uptake via clathrin- and caveolae-mediated endocytosis, with subsequent lysosomal localization. Controlled PTX release can be synergistically triggered by acidic pH and NIR-II laser irradiation, resulting in significant tumor inhibition in both in vitro and in vivo models [185]. Importantly, ferritin nanocages have recently been explored for boron neutron capture therapy (BNCT), a clinically emerging radiotherapeutic strategy. Apoferritin loaded with boron-rich clusters achieves high payload capacity, physiological stability, and receptor-mediated uptake via SCARA5, leading to significant tumor cell suppression upon neutron irradiation. These findings highlight apoferritin as a promising platform for radiotheranostics [186].

### 3.5. Transferrin

Representing a biologically validated yet receptor-constrained targeting strategy rather than a standalone nanomaterial platform, transferrin is an endogenous iron-transport glycoprotein extensively exploited in theranostic nanomedicine due to the overexpression of transferrin receptors (TfRs) in many cancer types [187]. Tf-based nanosystems enter cells via receptor-mediated endocytosis, thereby enhancing tumor accumulation and intracellular delivery.

This targeting strategy has been widely employed in the design of multifunctional nanoplatforms for near-infrared (NIR) imaging-guided photodynamic therapy (PDT) and photothermal therapy (PTT), Förster resonance energy transfer (FRET)-based monitoring of drug release, and MRI/NIR-guided combination therapies [187,188,189,190]. However, despite these advantages, several translational challenges remain, including variability in TfR expression among tumor types, competition with endogenous transferrin, and relatively rapid systemic clearance.

Several studies have demonstrated the versatility of Tf-functionalized systems. For instance, transferrin-based nanoparticles loaded with IR780 iodide have been developed using a “molecular switch” strategy to address the poor aqueous solubility and low tumor-targeting efficiency of this dye. These nanoparticles enabled NIR imaging and combined PDT/PTT upon single 808 nm laser irradiation, showing enhanced cellular uptake, high stability (up to 48 h), and a significant photothermal effect in CT26 cells and tumor-bearing mice [187].

Similarly, indocyanine green (ICG) has been loaded onto holotransferrin to construct a theranostic platform for fluorescence and photoacoustic dual-mode imaging-guided PTT in glioma models, further demonstrating the applicability of Tf in multimodal imaging approaches [188]. In another strategy, transferrin has been used as a nanotemplate to generate blue-emitting Cu nanoclusters (Tf-Cu NCs) co-loaded with doxorubicin (Dox). Upon UV excitation, FRET occurs from the Tf-Cu NCs (donor) to Dox (acceptor), resulting in red emission that enables real-time monitoring of intracellular drug release. This system has been validated using Dalton’s lymphoma ascites (DLA) models, highlighting its potential for simultaneous imaging and therapy [189].

Beyond fluorescence-based applications, Tf-functionalized magnetic nanoparticles have also been explored. Iron oxide nanoparticles (IONPs), known for their biocompatibility and suitability as MRI contrast agents, have been functionalized with transferrin to enhance tumor targeting. In one example, nanoparticles were further modified with TAT peptides containing nuclear localization signals and Cy7 fluorophores for real-time tracking. Subsequent conjugation of Tf to PEGylated surfaces yielded a multifunctional platform capable of nuclear targeting, MR/NIR imaging, and PTT. Upon NIR irradiation, this system exhibited significantly enhanced therapeutic efficacy in A549 tumor-bearing mice, particularly due to its ability to deliver therapeutic effects directly to the cell nucleus [190].

### 3.6. Additional Protein-Based Nanoplatforms

Beyond systems based on albumin, ferritin and transferrin, a wide range of protein-based nanoplatforms—including virus-like particles, protein–polymer hybrids and membrane-coated nanoparticles—have been developed with the aim of achieving functional modularity and biological mimicry. Whilst these approaches offer unprecedented design flexibility and biological sophistication, they also pose challenges in terms of manufacturing complexity, immunogenicity and regulatory translation, positioning them predominantly as advanced preclinical strategies.

Encapsulin derived from *Thermotoga maritima* has emerged as a versatile platform for drug delivery and the development of novel therapeutic strategies [24]. A representative study demonstrates the reprogramming of *T. maritima* encapsulin (TmEnc) into a targeted photodynamic therapy (PDT) nanocage. In this approach, the naturally bound riboflavin is first removed to generate apo-TmEnc, which is subsequently reconstituted with the photosensitizer rose bengal while preserving structural integrity. Surface functionalization is achieved through the insertion of the EGFR-targeting peptide AEYLR at two exposed positions, enabling selective uptake by EGFR-overexpressing cancer cells. Upon light irradiation, the engineered nanocages induce strong photocytotoxic effects, reducing HeLa cell viability to below 10%, whereas unmodified TmEnc remains non-toxic [25]. These findings establish TmEnc as a robust, low-cost, and modular protein nanocage suitable for targeted PDT and broader biomedical nanotechnology applications.

Beyond phototherapy, encapsulins have demonstrated potential as programmable nanoplatforms for enzyme delivery and enhancement. Genetic encapsulation of the tandem nitroreductase tdNfsB significantly improves enzyme stability, resistance to degradation, and catalytic efficiency, overcoming key limitations associated with conventional immobilization strategies. Moreover, pore engineering—supported by cryo-electron microscopy revealing multiple structural states—has emerged as a critical factor in optimizing substrate diffusion and overall nanoreactor performance. Importantly, this system shows strong relevance for anticancer therapy: encapsulated tdNfsB (Enc{tdNfsB}) enables efficient activation of prodrugs within tumor cells, including through extracellular mechanisms, thereby offering a promising approach for enzyme-directed therapies with reduced systemic toxicity [26]. Collectively, these results position encapsulins as powerful tools for robust biocatalysis and advanced therapeutic applications in oncology.

The therapeutic versatility of encapsulins has been further expanded through the development of fully genetically encoded, two-step in vivoligation-driven drug delivery systems for precision oncology. In this strategy, a HER2-targeting affibody fused to SpyCatcher is used for tumor pre-localization, followed by administration of doxorubicin-loaded encapsulins displaying SpyTag. This design enables high-affinity covalent assembly directly at the tumor site, addressing longstanding challenges related to nanoparticle reproducibility and inefficient tumor accumulation. The system achieves markedly improved therapeutic outcomes, including up to 95% tumor growth inhibition—outperforming both one-step delivery approaches and free doxorubicin—while eliminating cardiotoxicity [27]. These results highlight the potential of self-assembling protein nanoparticles to enable safer, more selective, and modular strategies for targeted cancer therapy.

These findings highlight their potential for precision oncology and theranostic applications.

#### 3.6.1. Virus-like Particles

Virus-like particles (VLPs) constitute a rapidly expanding class of protein-based nanocarriers for theranostic applications. These systems are characterized by genetically tunable architectures, high structural uniformity, and the ability to be functionalized chemically or genetically. These characteristics allow for the incorporation of imaging agents, therapeutic payloads and homing ligands into a single platform, although this multifunctional integration can introduce additional complexity into design and optimization.

Among these platforms, bacteriophage-derived VLPs such as Qβ and P22 have been extensively explored as multifunctional nanocarriers. Qβ-based VLPs have been explored as photocaged drug delivery systems, where doxorubicin molecules remain inactive until light-triggered release, enabling spatial and temporal control over chemotherapy. While these systems demonstrate that they can achieve high drug loading and precise activation, challenges related to aggregation, colloidal stability, and the need for external activation sources, including limited tissue penetration of light, remain significant barriers for clinical translation [191].

P22 VLPs possess a large and porous interior cavity that allows for high-density encapsulation of imaging agents and therapeutic cargos. In particular, P22 capsids modified with branched oligomer networks have achieved exceptionally high gadolinium payloads, resulting in high relaxivity MRI contrast agents. These studies highlight the potential of viral protein cages as imaging-oriented theranostic platforms, although the complexity of multistep chemical modifications and concerns related to immunogenicity may hinder scalability and regulatory translation [192].

Beyond passive or imaging-focused designs, modular P22 VLP systems have been developed using post-translational ligation strategies such as the SpyTag/SpyCatcher “bacterial superglue” approach. This approach allows for the covalent bonding of large targeting ligands, including affibody molecules specific for EGFR or HER2, while preserving capsid integrity. These target-tunable VLPs have demonstrated receptor-specific imaging and targeting cytotoxic effects via pH-responsive prodrug release, illustrating the capabilities of protein cages as highly modular and multifunctional theranostic platforms. However, these systems remain at an early translational stage due to manufacturing complexity and limited in vivovalidation [193].

In parallel, protein–polymer hybrid systems are emerging as complementary platforms, offering genetically encoded compartmentalization, enzyme encapsulation, and programmable cargo release. While these systems were not explored in detail here, they further expand the landscape of protein-based theranostics and underscore the growing importance of genetically encoded nanocontainers in precision nanomedicine.

#### 3.6.2. Protein–Polymer Hybrid Nanoplatforms

Protein–polymer hybrid nanoplatforms represent a biomimetic strategy in which biological functionality is introduced through molecular engineering. These systems typically combine synthetic polymers with functional proteins, enabling enhance control over physicochemical properties as size and stability, and allowing the introduction of stimuli-responsive behavior.

A representative example is the ROS-responsive polymeric theranostic system reported by Zhou et al. which integrates photodynamic therapy, multimodal imaging, and systemic immunotherapy through modulation of the mTOR signaling pathway. In this system, phototherapy-induced immunogenic cell death promotes dendritic cell maturation and CD8+ T-cell activation, resulting in suppression of both primary tumors and metastatic lesions [194,195].

#### 3.6.3. Cell Membrane-Coated Hybrid Systems

Unlike other approaches, cell-coated nanoplatforms represent a distinct biological interface strategy, in which the surface of the nanoparticles is repurposed to replicate higher-order cellular functions, rather than simple interactions between receptors and ligands [196]. Within this category, we can distinguish different solutions, depending on the functional origin of the membrane and the dominant biological function transferred to the nanocarrier. Thus, we have coatings derived from erythrocytes, which primarily confer immune evasion and prolonged circulation [195]; platelet membranes, which enable the recognition of vascular lesions and orientation towards tumor-associated endothelium [197]; tumor cell membranes, which introduce homotypic adhesion and antigenic mimicry [198]; and hybrid membranes that deliberately integrate complementary functions, typically combining immune evasion with active tumor recognition [199].

In this context, hybrid platelet–tumor cell coatings represent a rational evolution rather than an incremental modification, as they mechanically separate circulatory stability from orientation specificity and partially overcome the functional trade-offs observed in single-origin membranes [199]. Despite their compelling preclinical efficacy, membrane-coated systems also present significant translational limitations, such as batch-to-batch variability in membrane composition, limited scalability of membrane isolation and fusion processes, stability of protein orientation during storage, and poorly defined immunogenicity profiles following repeated administration [196,197]. It is important to note that, whilst these platforms excel in terms of biological mimicry and multivalent interactions, they do not intrinsically resolve the fundamental manufacturing and regulatory challenges associated with complex materials of biological origin [196]. Therefore, their realistic clinical value lies not in replacing established classes of nanocarriers, but in addressing specific bottlenecks in delivery—such as immune clearance, vascular adhesion or homotypic targeting [197,198].

#### 3.6.4. Comparative and Translational Perspective

From a translational standpoint, protein nanocages, protein–polymer hybrids, and membrane-coated systems represent distinct but complementary biomimetic strategies. Protein nanocages excel in structural precision and genetic programmability but face challenges related to immunogenicity and manufacturing complexity [191,192,193]. Protein–polymer hybrids offer superior scalability and chemical reproducibility [194,196], whereas membrane-coated systems provide unmatched targeting and immune modulation at the cost of increased biological variability and regulatory complexity [196,197,198,199].

Finally, if we were to place the protein-based nanoplatforms discussed in this section on a spectrum defined by clinical precedent, manufacturing reproducibility, and regulatory feasibility, we would find human albumin-based nanoparticles at one end, as they represent clinically benchmarked systems with established biocompatibility and relatively mature translational pathways (Table 1). Apoferritin- and transferrin-based platforms would occupy an intermediate position, combining biologically derived functionality with increasing, but potentially manageable, translational complexity. Lastly, we could consider the most structurally and functionally sophisticated systems—including elastin-like polypeptides, virus-like particles, protein–polymer hybrids, and membrane-coated nanoparticles—to be at the opposite end of the spectrum. These offer enhanced multifunctionality but still face limitations related to scalability, immunogenicity, manufacturing standardization, and regulatory translation. Despite their diverse architectures, these platforms collectively illustrate a central challenge in theranostic nanomedicine: balancing functional complexity with translational robustness and realistic manufacturing constraints.

To illustrate how these materials are translated into functional platforms, representative theranostic nanosystems based on different protein scaffolds are summarized in Table 1. The focus here is not on intrinsic material propertiesbut on specific implementations, including imaging modalities, therapeutic strategies, and design rationale underlying their multifunctional behavior.

Comparative evaluation of protein-based theranostic nanomaterials should rely on quantitative performance metrics rather than qualitative descriptions alone. Relevant endpoints include tumor inhibition rate, photothermal conversion efficiency, circulation half-life, tumor-to-normal tissue uptake ratio, cellular internalization efficiency, imaging signal enhancement, and drug loading capacity, which together provide a more robust framework for translational comparison [83,87,88,200]. This is particularly important because protein nanoplatforms differ not only in composition and targeting strategy, but also in pharmacokinetics, biodistribution, and therapeutic mechanism, all of which can strongly influence apparent efficacy [87,200]. Accordingly, the comparative analysis of the main protein-based systems can be guided by their most informative quantitative readouts rather than by structural complexity alone.

Among the most representative protein-based nanomaterials for theranostics, albumin, ferritin, transferrin, and silk fibroin occupy a central position because they combine biocompatibility, structural versatility, and strong potential for multimodal imaging and therapy [88,125,143,152]. Albumin-based systems frequently achieve high photothermal conversion efficiencies and strong tumor accumulation; for example, Gd_2_O_3_/CuS–albumin hybrid systems and related constructs have demonstrated efficient photothermal tumor ablation and complete tumor elimination in murine 4T1 models under NIR irradiation [106,107,108], while other albumin-based nanotheranostics incorporating IrO2 or similar components show high photothermal conversion efficiency and enhanced imaging-guided therapy [109]. Ferritin nanocages are notable for high loading capacity, receptor-mediated targeting, and strong tumor inhibition rates, with MnO_2_-Dox@HFn nanozymes reporting tumor inhibition values of up to 78.5% in vivo, together with enhanced MR imaging contrast [149], and other apoferritin systems enabling efficient cargo encapsulation through pH-triggered assembly with high loading efficiency and controlled release [146,147,148,149]. Similarly, ferritin-based constructs such as CuS-loaded apoferritin or dye-functionalized nanocages have demonstrated efficient tumor accumulation and strong photothermal or chemo-photothermal responses in glioma and carcinoma models [147,148]. Transferrin-functionalized platforms are particularly attractive for receptor-mediated targeting, showing enhanced cellular uptake, improved tumor accumulation, and sustained stability (up to ~48 h in physiological conditions in some IR780-loaded systems), although their performance may be limited by receptor saturation and faster clearance [152,153,154]. In parallel, silk fibroin systems exhibit efficient drug loading, tunable release, and significant tumor growth suppression in vivo, with ICG-loaded silk nanoparticles and MnO_2_-based composites demonstrating effective imaging-guided photothermal and combination therapy in glioma and breast cancer models [125,126,127]. Together, these systems illustrate that the optimal theranostic platform is not defined solely by multifunctionality, but by how efficiently it balances efficacy, targeting precision, pharmacokinetic behavior, and practical translational feasibility.

When comparing platforms, the most informative quantitative metrics are tumor inhibition rate (e.g., up to ~78–80% in ferritin-based systems), photothermal conversion efficiency (commonly >30–40% in optimized albumin/inorganic hybrids), tumor-to-normal tissue ratio (frequently >5–10 in receptor-targeted systems), and circulation half-life (typically several hours to days depending on the protein carrier), as these parameters directly capture efficacy, selectivity, and translational feasibility within a unified framework [83,87,149,200]. In addition, drug loading capacity (often reaching tens of wt% in protein nanocages) and imaging signal enhancement (e.g., increased MR relaxivity or NIR fluorescence intensity) help distinguish platforms with similar therapeutic outcomes but different delivery efficiency or diagnostic performance [88,143,146].

While Table 1 highlights representative applications, it does not allow for direct comparison between platforms in terms of performance. To address this limitation, Table 2 compiles quantitative metrics reported for selected nanosystems.

Importantly, these quantitative parameters should not be interpreted in isolation, as differences in experimental models, tumor types, and administration routes may influence reported values. Nevertheless, when considered alongside the structural features (Table 3) and functional design (Table 1), they offer valuable insights into the relative advantages and limitations of each platform from a translational perspective.

#### 3.6.5. GMP Manufacturing Challenges

While theranostic applications emphasize combined diagnostic and therapeutic functions, drug delivery systems remain the most clinically advanced implementations of protein-based nanomaterials.

The GMP-compliant manufacturing of multifunctional hybrid nanoplatforms remains a major bottleneck for clinical translation, largely due to their intrinsic structural complexity and multicomponent nature. Unlike conventional small-molecule drugs, nanomedicines are characterized by multiple interdependent critical quality attributes (CQAs), including particle size, morphology, surface charge, and drug loading efficiency, which must be simultaneously controlled within narrow ranges to ensure safety and efficacy [202]. This multidimensional quality framework significantly complicates process validation and standardization under GMP conditions.

A key challenge lies in achieving batch-to-batch reproducibility, which is essential for regulatory approval. Variability in raw materials, sensitivity to minor changes in process parameters, and the multistep nature of nanoparticle fabrication can all lead to significant inconsistencies in physicochemical properties and biological performance [203]. In particular, small variations in mixing conditions, temperature, or solvent composition during scale-up can result in substantial deviations in particle size distribution or drug encapsulation efficiency, ultimately affecting therapeutic outcomes [204]. These issues are especially pronounced in hybrid nanoplatforms, where the integration of organic and inorganic components introduces additional sources of variability and complexity [45].

Scale-up from laboratory to industrial production represents another critical hurdle. While many nanoplatforms can be reproducibly prepared at small scale, their manufacturing processes are often difficult to translate to large-scale production while maintaining consistent quality attributes. This challenge is largely attributed to the high sensitivity of nanoparticle formation mechanisms, such as nucleation and self-assembly, to slight variations in processing conditions, which can lead to deviations in size, morphology, and functional performance. Consequently, ensuring scalability without compromising reproducibility remains a fundamental challenge for GMP implementation and industrial translation [205].

From an analytical and quality control perspective, the characterization of multifunctional nanoplatforms is significantly more complex than that of conventional pharmaceuticals. In addition to chemical composition, comprehensive physicochemical characterization is required to assess parameters such as particle size distribution, surface properties, and structural integrity, often requiring advanced and non-standardized analytical techniques. The lack of harmonized analytical protocols and standardized regulatory frameworks further complicates comparability assessments and quality control across production batches.

Regulatory challenges are further exacerbated by the hybrid and multifunctional nature of these systems, which often combine therapeutic, diagnostic, and targeting functionalities within a single platform. This convergence blurs the boundaries between drugs, biologics, and medical devices, leading to uncertainties in regulatory classification and approval pathways. Moreover, the absence of universally harmonized guidelines for nanomedicines continues to hinder their global clinical translation and industrial deployment.

Finally, the increased complexity of multifunctional hybrid nanoplatforms has direct implications for manufacturing cost and feasibility. The use of high-purity materials, sophisticated fabrication techniques, and multistep assembly processes contributes to elevated production costs, while stringent quality control requirements further increase operational burden. As a result, despite their promising therapeutic potential, many hybrid nanoplatforms remain difficult to translate into clinically viable products. These considerations highlight a central challenge in nanomedicine development: balancing functional sophistication with manufacturability, favoring simpler and more robust systems that are compatible with GMP requirements and scalable production processes [206,207].

## 4. Protein-Based Nanodevices for Drug Delivery

**Figure 3:**
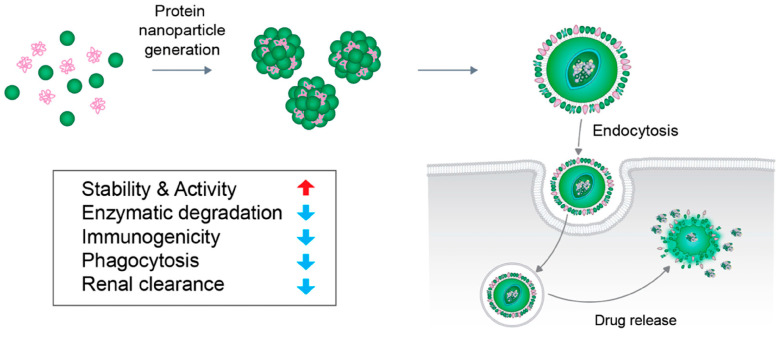
Scheme of protein nanoparticle uptake and drug release. Protein nanoparticles are internalized via endocytosis and, once inside the cell, insoluble drugs are released. Protein nanoparticles present increased stability and protection from enzymatic degradation, immune response, phagocytosis, and renal clearance, thereby achieving increased half-life of the drug. Reproduced with permission from Hong et al. [208].

As discussed previously, protein-based nanomaterials represent a cutting-edge frontier in targeted drug delivery due to their exceptional biocompatibility, biodegradability, and low toxicity. Fabricated from natural mammalian proteins (elastin, nidogen, gelatin, albumin or engineered silk), microbial toxins or virus nanocages (Figure 3), these smart nanocarriers offer high versatility because their sequence and structure can be precisely controlled. Moreover, these nanomaterials can be chemically modified to encapsulate diverse therapeutic agents and protect them from premature degradation. In this section, we describe protein nanocarriers specifically designed for drug delivery purposes, including specific cellular stimuli (pH levels, enzymes, or temperature changes), to achieve precise, controlled drug release directly at the target site. Consequently, they maximize therapeutic efficacy while significantly reducing systemic side effects, offering a highly promising platform for personalized medicine and advanced cancer therapies.

### 4.1. Albumin

Many of the first nanocarriers obtained from natural proteins were based on albumin, whose presence has been found to be critical in the cellular uptake process. Indeed, to date, the only protein nanocarriers in clinical use are based on albumin nanoparticles. HSA has been successfully used in Abraxane^®^, a nanomedicine approved by both the European Medicines Agency (EMA) and the United States Food and Drug Administration (FDA). These albumin-based NPs loaded with paclitaxel are used clinically in the treatment of metastatic breast cancer, metastatic pancreatic adenocarcinoma, and non-small-cell lung cancer [202,209]. Results showed significant improvement of prolonged disease control, as well as overall survival, progression-free survival, and response rate. However, its clinical application is limited by global yield of HAS, which could be solved by developing recombinant HSA (rHSA) as a biocompatible albumin replacing the HSA extracted from human blood [210].

In triple-negative breast cancer, Yuan et al. compared the action of paclitaxel nanoparticles bound to albumin (Abraxane^®^) with micellar Taxol, showing that although the concentration of Abraxane^®^ in blood was three- to five-fold lower than Taxol, it was more efficient in terms of drug delivery and intracellular uptake as similar drug tumor concentration and 10-fold higher tumor/plasma ratio were achieved in SUM149 xenograft NOD/SCID mouse model. However, its efficacy in reducing tumor size was similar in breast cancer xenografts in NOD/SCID mice. Nonetheless, this albumin nanoparticle may be used against metastatic diseases, as it is much more effective than Taxol in metastatic diseases, significantly reducing breast CSCs [211].

The same formulation of albumin-bound paclitaxel nanoparticles was used in a clinical trial in non-small-cell lung cancer (NSCLC). This phase III clinical trial enrolled patients with NSCLC previously treated with cytotoxic chemotherapy [56]. The results supported the use of Abraxane^®^ as a standard treatment option for patients previously treated with cytotoxic chemotherapy due to its efficacy and tolerability. Also, results from a phase III clinical trial conducted in patients with NSCLC led Spigel et al. to propose four 21-day cycles of 100 mg/m^2^ nab-paclitaxel (nanoparticle albumin-bound paclitaxel) as an alternative maintenance treatment [212]. Takeuchi et al. designed a randomized phase II trial to determine the optimal dose, activity, and safety of weekly administered nab-paclitaxel in previously treated patients with advanced NSCLC. This study concluded that both the standard dose (100 mg/m^2^) and the lower dose of nab-paclitaxel (70 mg/m^2^) demonstrated activity in NSCLC patients [213]. Therefore, the lower dose of nab-PTX (70 mg/m^2^) would be more reasonable as therapeutic option.

A thorough understanding of absorption mechanisms in specific cancer types is essential for designing multi-receptor-targeted delivery systems. Stukan et al. highlighted HSA-based nanoparticles as broadly effective tools presenting the potential to overcome multidrug resistance and improve the specific targeting of cancer cells. Thus, deeper insights into cancer cell uptake are key to achieving enhanced therapeutic efficacy [206], since the current lack of tumor specificity does not solve the problem of adverse side effects in healthy tissues. Responding to the need to find increasingly effective and precise anticancer therapeutic strategies, new-generation protein nanodevices have been designed for active and site-specific targeting.

### 4.2. Elastin-like Polypeptides (ELPs)

Although albumin is the gold standard for nanotherapeutics translated into clinics, other natural proteins have been explored to build smart scaffolds. Elastin, as an important element within the ECM, has been widely explored in regenerative medicine due to its great biocompatibility and biodegradability. Among protein-based nanomaterials, recombinant elastin-like polypeptides are inherently stimuli-responsive nanomaterials with wide applicability in intracellular targeting and drug delivery [207]. These protein-based nanocarriers also offer controlled synthesis and reproducibility [74].

With regard to anti-tumor strategies, doxorubicin (Dox) is one of the most commonly selected therapeutic molecules in drug delivery nanosystems for both preclinical trials and commercial formulations. For example, Ryu et al. designed a drug delivery system based on an ELP scaffold (60 kDa) to deliver a 6-maleimidocaproyl amide derivative of Dox to breast cancer cells [214]. The authors included a matrix metalloproteinase (MMP)-cleavable sequence (PLGALG) and the cell-penetrating peptide (CPP) Tat in the construct to take advantage of an enzyme typically located within tumor tissues as an external stimulus. It is worthy to highlight the design of this nanosystem, in which the cargo (CPP-Dox) is one-twentieth that of the parental ELP construct (60 kDa), thereby improving dox pharmacokinetics, with quick infiltration into tumor cells and reduced clearance. Thus, the system was specifically designed to release the CPP–drug complex, which showed significantly enhanced cellular uptake and cytotoxicity in Dox-sensitive cancer cells (MCF-7 and MES-SA) compared to undigested ELP-CPP-Dox. Interestingly, incubation of Dox-resistant breast cancer cells (NCI/ACR and MES-SA/Dx5) with the cleavable nanosystem resulted in almost twice the cytotoxicity as free Dox or undigested ELP-CPP-Dox. Even though this study only determined the effect in vitro, these results could open up a route for improving the efficiency of drug delivery systems in the future. Moreover, this study demonstrates ELPs’ potential in multiple-targeting anti-cancer strategies.

Another elastin-based system for delivering Dox was explored by Dragojevic et al. [215]. This construct consisted of an ELP scaffold (60 kDa), the cell-penetrating peptide SynB1, a pH-sensitive cleavable linker, and Dox. In particular, attachment of Dox to ELP was achieved by two biochemical linkers, an acid-cleavable hydrazone, and an amino acid sequence recognized by lysosomal enzymes (GFLG). A comparison between non-cleavable Dox and cleavable Dox in MCF-7 human breast cancer cells and NCI/ADR human ovarian Dox-resistant cancer cells showed different intracellular localizations. Furthermore, the cleavable construct triggered apoptosis at a lower rate than the non-cleavable system. These results suggest a different mechanism of action for non-cleavable Dox compared to free Dox. Moreover, the in vitro results demonstrated that cleavable Dox had a similar cytotoxic effect to free Dox in MCF-7 Dox-sensitive cells and a lower efficiency in Dox-resistant cancer cells. In contrast, non-cleavable Dox exhibited significantly lower cytotoxicity in both cell lines. IC50 assays indicated that non-cleavable Dox only required a 4-fold higher concentration to achieve the same toxicity in Dox-resistant cells compared to MCF-7 Dox-sensitive cells, whereas free Dox required a 100-fold concentration increase. Thus, although the SynB1-ELP-GFLG-Dox construct showed lower anti-tumor potential in vitro than free dox, the protein nanoconstruct could offer notable advantages, including improved accumulation within tumors and longer plasma half-life. Also, this study suggests that the non-cleavable conjugation of Dox to an elastin-based scaffold provides potential advantages in Dox-resistant breast cancer cells, even triggering a different mechanism of action. This difference may be mainly explained due to the molecular weight of the SynB1-ELP-GFLG-Dox construct (60 kDa), which requires endocytosis-mediated entrance, instead of the free diffusion of small doxorubicin. In another study, the same authors explored the use of the construct SynB1-ELP–Dox as a thermally responsive nanocarrier in MDA-MB-231 xenografts in nude mice when the temperature is increased above 37 °C [216]. Although free Dox (10 mg/kg) showed lower accumulation in the kidneys, the thermally targeted nanosystem showed a two-fold higher tumor uptake and a fivefold decrease in the heart compared to the free drug and the thermally non-responsive control, thereby suggesting that thermal targeting enhances tumor accumulation and minimizes cardiotoxicity. Thus, ELP-Dox conjugates not only provide a thermally targeted drug delivery strategy, but also overcome multidrug resistance in cases with difficult successful rates.

Sarangthem et al. explored the antitumor effect of self-assembled ELP micelles carrying the pro-apoptotic peptide KLAKLAK, which specifically targets the interleukin-4 receptor (IL-4R) via the AP1 peptide [217]. These micelles self-assembled at physiological temperature into nanoparticles of 110~130 nm (ELP-KLAK) or 180~210 nm (AP1-ELP-KLAK). Therefore, the presence of the AP1 peptide significantly affected the physical characteristics of the resulting nanosystem. Results showed that the targeted nanosystem induced caspase 3-mediated apoptosis in vitro, and intravenous administration of the AP1-ELP-KLAK construct diminished tumor growth in a glioblastoma mouse model. This approach may be a suitable tool for non-invasive imaging, enabling real-time monitoring of antitumor effects in animal models with tumor cells that express stable reporter systems. However, additional studies in orthotopic models are needed to test the accuracy of the targeted ELP micelles. Therefore, non-invasive imaging approaches using biosensors may provide potential tools for real-time evaluation of cell apoptosis explored in cancer therapy.

Thomas et al. conjugated a peptide that is able to inhibit NFκB activation (p50) to a thermoresponsive ELP nanocarrier (63 kDa) [218]. The resulting protein-based polymer was shown to self-assemble at 40.3 °C by measuring the maximum OD at 350 nm, so that the polymer aggregated after application of mild hyperthermic conditions (41 °C). The SynB1 cell-penetrating peptide was conjugated to the N-terminus of ELP to specifically induce internalization into breast cancer cells, where p50 induced apoptosis by blockade of the intranuclear import of NFκB, thereby inhibiting cell proliferation. SynB1-ELP1-p50 aggregates were found to be localized in the cytoplasm and along the nuclear periphery. As expected, treatment with SynB1-ELP1-p50 in combination with hyperthermia for 7 days improved the cytotoxic effect compared to treatment at physiological temperature (37 °C). Thus, the authors showed the advantages of protein ELP carriers for delivering therapeutic peptides in a controlled manner. In particular, this work demonstrates the advantages of inhibition of the NFκB signaling pathway to inhibit the growth of mammary cancers. Also, the inhibitory peptides of NFκB may be explored to improve the sensitization of cancer cells to traditional therapies such as chemotherapy or ionizing radiation, in order to diminish drug resistance and improve treatment outcomes.

An ELP polymer was specifically designed to target cisplatin-resistant melanoma through the incorporation of 16 copies of M-peptide, a Cis-R melanoma-targeting ligand (M-peptide) [219]. This ELP (M_16_E_108_) contained 16 targeting ligands and self-assembled at 39 °C into aggregates with a diameter of 1.8 µm. The size of the construct was precisely designed to avoid the obstruction of blood vessels after systemic administration. Cis-R B16F10 melanoma cells showed enhanced accumulation of M_16_E_108_ compared to non-cisplatin-resistant B16F10 cells and an ELP control lacking M-peptide (E147). Moreover, the results demonstrated higher accumulation in Cis-R melanoma tumors than B16F10 tumors after intravenous administration in mice. In all cases, only minimal accumulation was detected in mice treated with E147 aggregates. Therefore, the study showed the successful biodistribution and accumulation of the targeted protein-construct M_16_E_108_ in Cis-R melanoma both in vitro and in vivo. Thus, M_16_E_108_ presents remarkable potential to design novel approaches against melanoma cells’ drug resistance towards cisplatin.

Gonzalez-Valdivieso et al. reported the first study testing the therapeutic potential of elastin-based nanoparticles as drug delivery systems in clinically relevant pancreatic cancer patient-derived models [220]. In this work, the authors tested previously described ELR polymers that are able to self-assemble into advanced nanocarriers (70 nm and negative ζ-potential) with high conformational complexity [221] to deliver an Akt peptide inhibitor in 2D and 3D in vitro models. The nanoparticles included different bioactive sequences to facilitate their internalization (LAEL peptide), enzymatic release of the inhibitor mediated by endolysosome protease Cathepsin D, and escape from the endo-/lysosomes at acidic pH to reach the target within the cell cytoplasm. The nanocarriers were shown to dramatically affect cell viability and metabolism by inhibiting the Akt signaling pathways, thereby blocking NF-κB-controlled cell survival and unlocking caspase 3- and JNK-mediated apoptosis. Moreover, this work highlighted the safety after systemic administration in mice, since the nanocarriers presented a long circulating half-life in blood (5.3 h), and histopathological examination revealed no major effects on the microstructure or physiology of vital organs, specifically the heart, liver, spleen and kidney. Following a similar amphiphilic ELP scaffold, Gonzalez-Valdivieso et al. combined recombinant DNA technology and the supercritical antisolvent (SAS) technique to develop smart nanohybrids carrying the Akt inhibitor and encapsulated DTX to specifically release both anti-tumor drugs inside colorectal cancer (CRC) cells in vitro and in vivo. The design was completed by including a DNA aptamer to selectively target cells expressing CD44, a membrane receptor overexpressed in CRC. This 53 nm dual-approach nanosystem was found to selectively reduce cell viability (2.5% survival in colorectal cancer cells vs. 50% survival in endothelial cells), triggering both apoptosis- and necrosis mechanisms. In vivo assays in a colorectal cancer mouse model showed that these dual-approach nanohybrids significantly reduced the number of tumor polyps along the colorectal tract. Moreover, systemic administration of loaded nanohybrids improved the morphology of gastrointestinal crypts and recovery of tissue architecture. Thus, this therapeutic nanosystem opens up a promising approach for controlled release of combined drugs against a wide range of solid tumors [222]. Moreover, this combined strategy based on nanohybrids loaded with two different therapeutic agents triggering multiple mechanisms of action improves upon the results achieved by ELP nanoparticles only carrying one type of anti-cancer therapy.

In a recent study, Kelly et al. reported the combination of brachytherapy (131I) and immunotherapy (CpG oligodeoxynucleotide immunostimulant) in an injectable ELP system for sustained intratumoral delivery [223]. To that end, the system included an oligolysine tail (K12) capable of forming an electrostatic complex with CpG (ELP-K12/CpG). The developed ELP system self-assembled into particles of 137 nm of diameter above 33 °C. The preclinical study compared the ability of that complex to control tumors as a monotherapy and in combination with brachytherapy (131I-ELP). The ELP-K12 nanosystem significantly prolonged retention within the tumors (>21 days) compared to soluble CpG (<24 h). Furthermore, ELP-K12 increased the cellular uptake of CpG by activating the toll-like receptor 9 (TLR9). An ELP-K12/CpG depot was able to reduce tumor growth and lung metastasis burden in a breast cancer model, which contrasts with the lack of effect of CpG alone. However, the combination of brachytherapy (131I-ELP) and immunotherapy (ELP-K12/CpG) improved mouse survival since it avoided tumor growth and the development of lung metastases. Thus, the authors showed the outstanding potential of ELP depots as a promising therapeutic strategy for combined local delivery of radiotherapy and immunotherapy in poorly immunogenic, metastatic tumors.

In addition to the efficacy of drug delivery systems in cancer cells, another relevant parameter is their safety within the whole organism. In this context, Peddi et al. determined the nanotoxicology of ELP nanoparticles [224] loaded with rapamycin, whose encapsulation within the nanoparticles increased rapamycin’s solubility, as previously described in MDA-MB-468 breast cancer xenografts [225]. In vitro assays in HepG2 cells showed that nanoparticles measuring 46 nm with low polydispersity index (0.03) were internalized via endolysosomes and triggered cellular oxidative stress as a result of reactive oxygen species (ROS), which was not the case for empty nanoparticles or free rapamycin. As expected, rapamycin remained soluble and monomeric below the Tt (at 15 °C) as the ELP nanoparticles were unassembled. Antitumor efficacy and toxicology were determined in an orthotopic murine model of hormone receptor positive (HR+) breast cancer. Regarding the therapeutic effect, the results showed that rapamycin-containing nanoparticles suppressed tumor growth and the mTOR-signaling pathway. On the other hand, drug-loaded nanoparticles did not induce an immune response, such as hemolysis, complement activation, plasma coagulation, or platelet aggregation. It is important to highlight that, although cell internalization of loaded nanoparticles increased oxidative stress in vitro in comparison to free rapamycin or empty nanocarriers, no hepatotoxicity was observed in vivo after administration for one month. This could be explained by assuming that the critical concentration for ROS generation in the liver was not reached. As such, self-assembled ELP nanoparticles are shown as accurate vehicles to deliver rapamycin in safe and accurate drug delivery systems due to their modular coassembling.

ELP-based nanocarriers were also chosen to perform a toxicology study in mouse tumor models, focusing on renal functionality [226]. The ELP-VEGF chimeric protein (74 kDa) had been previously reported to successfully induce angiogenesis in a swine model of chronic renal disease, thereby improving renal vascular density, renal fibrosis, and renal function [227,228]. However, the high vascular permeability induced by VEGF therapy may trigger off-target toxicity and severe adverse effects in patients with solid tumors. ELP-VEGF administration in rats by bolus intravenous injection (0.1 to 200 mg/kg) did not provoke histopathological signals or changes in renal injury markers in plasma or urine. However, high doses (100 and 200 mg/kg) caused acute hypotension, increased the glomerular filtration rate, and decreased renal microvascular density after 14 days. Low doses (0.1–10 mg/kg) were also tested in a breast cancer mouse model. Although the results ruled out any effect on tumor growth rate or tumor mass, ELP-VEGF significantly increased the tumor vascular density in a dose-dependent manner. Although the therapeutic dose range failed to induce toxicity in healthy subjects, further assays are needed to better understand the effect of ELP-VEGF in cancer patients before preclinical testing of ELP-VEGF against kidney diseases [228]. Therefore, the development of ELP-VEGF therapies may provide the basis for more studies involving more models of acute and chronic kidney diseases.

A different approach involving the use of ELPs was designed to improve melanoma immunotherapy. Thus, Chen et al. developed an in situ-forming ELP-based chemically crosslinked hydrogel for the co-delivery of PD-L1 antibodies and gemcitabine, targeting immune checkpoints [229]. The ELP hydrogels showed slow degradation as well as sustained release over 20 days. In C57BL/6 mice, this system promoted CD8^+^ T-cell infiltration within the tumors (3.0-fold), enhanced antitumor immunity (60% Tregs depletion), and significantly extended survival. Therefore, these results show the promising potential of ELP hydrogels to deliver immune checkpoint inhibitors (ICIs) and chemotherapeutic agents to improve cancer immunotherapy. Even though elastin is a promising source with high tunability for specific target cells and tissues, their recombinant origin has been seen as a challenge to overcome. Thus, different proteins are also reported to develop protein-based nanodevices suitable for drug delivery purposes.

Therefore, we can conclude that encapsulins are a promising source for the design and development of drug delivery systems. However, further research is needed to overcome their important limitations when compared to other protein-based nanosystems already stablished in clinical trials or even clinically approved protein-based nanosystems.

### 4.3. Apoferritin

Apoferritin is a versatile protein widely used for when designing nanocarriers for controlled drug delivery [230]. Apoferritin has been recently explored to act as a bio nanoreactor, taking advantage of its 8 nm internal cavity and its pH-dependent reversible self-assembling ability, to direct the formation of inorganic nanoparticles of controlled size [231]. Multiple studies show that apoferritin offers a confined environment capable of encapsulating and stabilizing chemical molecules, thereby preventing aggregation and enabling stimulus-mediated release [232,233]. Furthermore, apoferritin present excellent biocompatibility, biodegradability, and surface functionalization, essential characteristics for the development of targeted drug delivery systems. Moreover, the achievement of precise control through electrochemical and bioelectrochemical stimuli supports the use of apoferritin as an interesting platform to encapsulate, transport and release drugs in a controlled manner, opening the door to smart apoferritin-based nanotherapies based on local stimuli [231].

Apoferritin has been explored in the treatment of Alzheimer’s disease, a major challenge due to the limited effectiveness of treatment focused on the central nervous system [234,235]. One example presents an apoferritin-based nano transporter (OAF) loaded with oridonin, a bioactive ent-kaurane diterpenoid triggering anti-inflammatory activity, cell cycle arrest, apoptosis induction, and angiogenesis suppression [236]. The construct included a dual targeting mechanism of the brain and liver via TfR1, aiming at the elimination of Aβ and reducing neuroinflammation and oxidative stress [88].

Beyond neurodegenerative diseases, apoferritin-based nanocarriers have also shown considerable promise in cancer therapy. A recent study on drug delivery proposes a Ca/DHA@AFn construct, which integrates two highly effective cell death modalities (ferroptosis and calcicoptosis) into a single system. The construct was based on biomineralized apoferritin nanoparticles capable of transporting dihydroartemisinin and Ca^2+^, whose release in the acidic tumor microenvironment triggers lethal calcium overload. This architecture overcomes one of the typical limitations of ferroptosis, such as the low accumulation of reactive oxygen species (ROS) within tumors, achieving a synergistic effect that enhances tumor cytotoxicity [237].

Similarly, other studies have explored the use of horse spleen apoferritin as a nanocarrier for metallodrugs in cancer treatment. A study evaluated horse spleen apoferritin as a nanocarrier to improve the efficacy and selectivity of an organo-rutenoid complex with anticancer activity [238]. The Ru-apoferritin complex exhibited sustained release over 6 days without the initial burst release. Therefore, this approach significantly improved drug availability compared to free forms of any metallic drugs [239]. In vitroAssays of MCF-7 breast cancer cells demonstrated that encapsulation increased the stability of the complex and selective cytotoxicity. Overall, the study confirms that apoferritin is an effective and biocompatible nanocarrier for metallodrugs, capable of modulating their release, mechanism of action, and tumor selectivity [238].

### 4.4. Encapsulins

Bacterial encapsulins have recently emerged as a new class of protein nanocapsids for targeted drug delivery [240]. In particular, the encapsulin from *Thermotoga maritima* was reengineered to display DARPin9.29, a ligand with high affinity for HER2, on its surface and to encapsulate the photosensitizer miniSOG as a cytotoxic cargo. The system self-assembles in *E. coli*, maintaining a stable, modular architecture. The resulting nanoparticles showed highly specific binding to HER2+ breast cancer cells. Also, upon light activation, this system generates ROS and, consequently, triggers apoptosis-mediated cell death, demonstrating efficacy and specificity [100]. Thus, these findings establish encapsulins as versatile and scalable platforms in which both surface ligands and internal therapeutic cargos can be exchanged to design tailored targeted therapies.

Further advances in encapsulin engineering have expanded their functionality for intracellular protein delivery. Thus, Kwon et al. recently presented T4 encapsulin (QtEnc), showing an exceptional permeability, which allows for the loading of proteins after assembling into protein nanocapsids. This feature enables rapid, modular, and wide-ranging loading, from small proteins to 482 kDa complexes, as well as multiplexed co-encapsulation. Based on the QtEnc encapsulin, a novel nanocarrier, QtEncNC, was developed for pH-dependent release and endosomal escape, achieving functional cytosolic delivery of therapeutic protein BLF1 in HeLa cells [241]. These results present QtEncNC as a versatile platform for intracellular protein delivery, with potential applications in cancer, gene editing, immunotherapy, and enzyme therapies.

Beyond drug and protein delivery, encapsulins have also been engineered as nanoreactors for oxidative cancer therapies. Aiming for the development of an encapsulin-based nanoreactor, the enzyme pyridoxine 4-oxidase (PNOX) has been shown to be successfully encapsulated inside the *Myxococcus xanthus* encapsulin, taking advantage of its natural self-assembly ability and CLP-mediated targeted cargo loading. PNOX generates hydrogen peroxide, thereby making this system an attractive platform for oxidative cancer therapies. This work stands out for its pore-engineering strategy, in which pore diameter is increased, together with improved substrate and product diffusion. After demonstrating notable increases in catalytic activity, the pore architecture is highlighted as a key factor in nanoreactor performance. Importantly, encapsulation provided high stability, with activity higher than 90% after 4 h at 37 °C [242]. This strategy demonstrates that encapsulins can be reprogrammed into efficient, stable, and scalable nanoreactors capable of generating ROS in a controlled manner, therefore positioning them as promising platforms for targeted oxidative therapies and advanced biotechnological applications.

Another biomedical application of encapsulin-based drug delivery systems is tumor imaging and real-time monitoring. Thus, a recent work by Milovanova et al. demonstrated that the encapsulin from *Quasibacillus thermotolerans* can function as an intrinsic magnetic reporter for tracking tumor cell dynamics. By modifying the glioma cells to biosynthesize biogenic magnetic nanoparticles within Qt shells, the study provides a sensitive alternative to conventional imaging approaches, thereby leveraging the nonlinear MPQ detection method to monitor signal changes during rapid cell proliferation. Overall, the study presents encapsulin-derived magnetic reporters as a powerful yet context-dependent tool for high-sensitivity tumor monitoring, complementing and refining existing imaging modalities in glioblastoma research. However, although encapsulins show promising results, limitations are still considerable: while Qt encapsulins enable robust iron sequestration and strong magnetic contrast, the magnetic signal decays within days as tumor cells divide, underscoring the challenges of applying nanoparticle-based tracking to fast-growing malignancies [243].

### 4.5. VLPs

Virus-like particles and nanocages are other examples of protein nanocarriers that have been explored for drug delivery approaches against cancer [108]. Although their viral origin could be seen as a limitation, VLPs present important advantages such as the lack of infectious ability. Moreover, VLPs can be produced in a wide variety of systems, such as mammalian cells, plant cells, insects, and bacteria [108]. In this context, Lieser et al. engineered E2 nanocages decorated with the SpyTag peptide on the surface, as well as the 4GE11-mCherry-SpyCatcher (SC) targeting system, for selective recognition of the epidermal growth factor receptor (EGFR) [244]. The E2 nanocage consists of 60 monomer subunits from the pyruvate dehydrogenase complex of the thermophilic bacterium *Bacillus stearothermophilus* and forms 25 nm nanocarriers that have previously been described for Dox encapsulation and delivery. Engineered nanocages decorated with 10% 4GE11-mCherry-SC were able to encapsulate up to 150 molecules of Dox and release almost 60% of their cargo during the first three hours under acidic conditions (pH 5). Furthermore, in vitro assays showed enhanced internalization (threefold) in inflammatory breast cancer (IBC) SUM149 cancer cells overexpressing EGFR compared to healthy MCF-10A breast epithelial cells with basal levels of EGFR. The improved uptake of targeted nanocages carrying Dox correlated with improved cytotoxicity in IBC cells, since nanomolar concentrations of the nanocages decreased cell viability to 56% after treatment for 48 h. More importantly, cell viability was significantly higher (78%) in MCF-10A cells under identical conditions. Therefore, VLPs are highlighted as tunable vehicles, since their nature triggers stimulation of both humoral and cellular immune responses, as well as strategies based on loading with immune-modulators. These features make VLPs an interesting source for protein-based nanodevices and have been studied in multiple clinical trials.

### 4.6. Other Protein-Based Nanocarriers

In addition to exploring new therapeutic molecules against cancer, some strategies have focused on new approaches to increase the solubility of drugs [245]. One of these studies was reported recently by Gleason et al. [246], who encapsulated the water-insoluble chemotherapeutic drug SN38 within protein-based micelle nanocarriers (23–25 nm) comprising a hydrophilic intrinsically disordered protein (IDP) domain derived from the human neurofilament heavy arm side chain protein bound to the hydrophobic tail 2Yx2A. Intracranial injection of the SN38-loaded micelles protected against neurological toxicity symptoms in healthy mice, while convection-enhanced delivery (CED) into mice bearing U251-MG glioblastoma multiforme (GBM) xenografts resulted in slower tumor growth and significantly increased survival compared with mice treated with the free drug or DPBS. These results highlight the potential of tailorable protein-based micelles to improve the delivery of hydrophobic chemotherapeutics to tumor tissues.

Peptide-based carriers have also emerged as promising drug delivery systems owing to their structural versatility, biocompatibility, and intrinsic bioactivity. In this regard, Xue et al. engineered a biomimetic peptide-based nanocarrier with regular round shape and 100 nm size by polymerizing L-glutamyl-L-lysine (albumin surface peptides) with L-phenylalanine (a cathepsin B–cleavable site) to encapsulate doxorubicin [247].

The albumin-derived shell prolonged systemic circulation and improved tumor penetration by retaining water molecules within the ECM of the tumor mass. Importantly, tumor-overexpressed cathepsin B (CB) selectively degraded the peptide scaffold, enabling controlled Dox release. In an MCF-7 tumor model, this nanodrug achieved a tumor inhibition rate of up to 97.2% while maintaining favorable biosafety [247].

The strategy of modulating the tumor microenvironment and eradicating ECM through the targeting and depletion of CAFs has been proposed as a novel therapeutic approach for desmoplastic solid tumors [248]. Given the pivotal role of CAFs in triple-negative breast cancer, where they facilitate tumor growth and promote angiogenesis, Wang et al. designed a nanoconstruct (spherical morphology with 190 nm diameter) decorated with CREKA peptide to specifically target fibronectin overexpressed in CAFs. This was achieved by conjugating hydroxyethyl starch–IR780 nanoparticles loaded with Dox to the peptide Cys-Arg-Glu-Lys-Ala. In 4T1 tumor models, tumor growth was significantly suppressed through the combined effect of chemotherapy and phototherapy. Overall, this strategy of tumor microenvironment modulation and cancer stem cell elimination via CAF targeting and depletion represents a promising therapeutic avenue for the treatment of desmoplastic solid tumors. Also, this study sets up the implications for the development of theranostic nanoparticles by tuning tumor mechanical properties.

A further example of engineered protein nanocarriers was reported by Alamo et al. [249]. Given that green fluorescent protein (GFP) is one of the most commonly used protein scaffolds presenting excellent biocompatibility, biological neutrality and stability, the authors developed a GFP-like structure containing the β-barrel G2 domain from human nidogen, whose structure is identical to that of GFP. Furthermore, the fusion protein included the T22 peptide, a potent ligand of chemokine receptor CXCR4 typically overexpressed in cancer cells, as a targeting system. The resulting protein was able to self-assemble into 10 nm nanoparticles with a negative surface charge (−9.13 mV). The nidogen-based protein scaffold was selectively internalized in HeLa cells via CXCR4 at a similar rate to the GFP-based construct and showed perinuclear localization. After in vitro characterization, the fusion protein was covalently linked to pentameric oligonucleotides of the genotoxic antimetabolite floxuridine (oligo-FdU). Incubation of CXCR4+ HeLa cells with the nidogen-based nanoconjugate resulted in significant cytotoxicity compared to free oligo-FdU, as well as a similar cytotoxic effect and IC50 as the GFP-based nanoconjugate (1.22 and 1.04 nM, respectively). Moreover, intravenous administration of the nanoconjugate in a CXCR4+ colorectal cancer mouse model resulted in high inhibition of tumor growth (−64.5%), characterized by a significant increase in cell death bodies and caspase 3 activation within the tumor tissue. However, the results showed that the nidogen-based nanoparticles carrying oligo-FdU had a broader therapeutic window than free oligo-FdU and their GFP counterpart. This is an example of engineered alternatives to exogenous proteins specifically designed to mimic both structural and functional properties of GFP while developing scaffolds for protein-based nanomedicines.

In addition to mammalian proteins, microbial toxins have also been used as protein scaffolds for drug delivery purposes. This source, together with the concept of self-assembling drugs using a molecule simultaneously as drug and vehicle, emerges as an original approach besides the difficulty of conventional chemistry to develop this kind of nanodevices. For example, the exotoxin A from *Pseudomonas aeruginosa* and the diphtheria toxin from *Corynebacterium diphtheria* have been used as self-assembling protein nanocarriers for oligo-floxuridine and monomethyl auristatin E (FdU and MMAE, respectively) [250]. T22-DITOX-H6 and T22-PE24-H6 nanosystems have been developed to include the T22 peptide, a selective ligand for the CXCR4 marker of ECM. In addition to the CXCR4-targeting peptide and a 6His tag, T22-PE24-H6 included the C-terminal KDEL motif to control intracellular trafficking. Both nanoconjugates presented a hydrodynamic size of around 50 nm, a negative surface charge, and high stability. With regard to the cytotoxic effect in CXCR4+ human cancer cells, simultaneous administration of unconjugated protein and chemical drugs (either T22-PE24-H6 and FdU or T22-DITOX-H6 and MMAE) showed a cumulative effect. However, this effect was not found when cancer cells were treated with the resulting nanoconjugates, which failed to improve the results of any of the components alone or the uncoupled drugs combined. Nevertheless, despite being less efficient, this strategy consisting of anticancer drug pairs (a tumor-targeted protein nanocarrier and an antiproliferative drug) may lay the foundation for minimizing pharmacological resistance to antitumoral drugs. This approach based on the combination of protein and non-protein drugs could be explored deeply aiming to reduce pharmacological resistance to antitumoral drugs, one of the main limitations of conventional chemotherapy.

Taken together, natural and recombinant protein-based nanomaterials exhibit distinct translational profiles, reflecting a balance between intrinsic biological compatibility, targeting capability, and controllable design. These differences highlight the importance of considering not only material properties, but also manufacturability, reproducibility, and clinical feasibility when evaluating their potential for translation.

Summary of intrinsic physicochemical features of nature-inspired synthetic/recombinant protein nanocarriers reviewed in this section is presented in Table 3. The table highlights relevant structural features, including domain organization and conformational properties, as well as key physicochemical parameters such as size, solubility, and surface charge. Additionally, the table relates these properties to their intended applications, providing a comparative overview that can guide the selection and design of protein-based nanomaterials for biomedical, environmental, and technological purposes.

**Table 3:** Intrinsic physicochemical and structural features of nature-inspired synthetic/recombinant protein nanomaterials reviewed in this section.

Proteins	Physicochemical Characteristics	Structure	Intrinsic Advantages	Limitations	Targeting Mechanism	Clinical Status	Refs.
**HSA**	High solubility; binds lipophilic molecules; MW ~65–70 kDa	Single polypeptide (~585 aa); α-helical, three domains	Long circulation half-life; molecular flexibility; endogenous origin	Limited drug loading (charge-related); conformational instability	Passive accumulation (EPR); gp60-mediated transcytosis; SPARC interaction	Clinically validated (Abraxane^®^, Fyarro^®^); extensive clinical use	[251,252,253,254]
**Silk**	High mechanical strength; low aqueous solubility	β-sheet crystalline structure; Gly/Ala/Ser-rich	Structural robustness; tunable degradation	Oxidation and enzymatic degradation	Mainly passive; ligand-mediated functionalization possible	Preclinical (nanoparticles); bulk material approved	[63,64,141,255]
**ELP**	Thermoresponsive; reversible phase transition; elastic	Repetitive pentapeptides (VPGXG); disordered, self-assembling	Precisely tunable phase behavior; recombinant design control	Sensitive to temperature/pH; aggregation at high concentration	Stimuli-responsive self-assembly (temperature/pH); customizable targeting motifs	Early preclinical	[64,245,256]
**Ferritin** **[149,200]**	Highly stable across pH and temperature	24-subunit nanocage (~12 nm; ~8 nm cavity)	Defined nanocage architecture; genetic modifiability	Limited endogenous loading; distribution in RES organs	Receptor-mediated (TfR1/CD71; SCARA5 subtype-dependent)	Phase I (vaccines); drug delivery preclinical	[97,257,258,259]
**Transferrin** **[149,201]**	Iron-binding glycoprotein; moderate stability	Single-chain (~679 aa); non-self-assembling	Natural receptor recognition (TfR1)	Competition with endogenous transferrin; off-target uptake	TfR1-mediated endocytosis	Preclinical–early clinical	[260,261,262]
**Encapsulin**	Highly stable nanocompartment; pH/temperature resistant	Self-assembled cages (20–45 nm)	Precise cargo encapsulation via peptide tags; modular assembly	Non-human origin; immunogenicity; limited PK data	Engineered targeting (peptides/affibodies)	Early preclinical	[100,101,103,263]
**Virus-like particles (VLPs)**	Highly ordered nanoscale assemblies (20–200 nm); biodegradable	Self-assembled viral capsids; symmetric, repetitive	Precise architecture enabling multivalent display	Intrinsic immunogenicity; pre-existing immunity; manufacturing complexity	Passive and engineered targeting; multivalent uptake	Clinically validated (vaccines); oncology applications preclinical	[108,109,114,264]

## 5. Conclusions

Nanomedicine has significantly expanded the landscape of cancer diagnosis and therapy by enabling improved drug delivery, targeting strategies, and the integration of therapeutic and diagnostic functionalities. However, despite extensive preclinical development, the clinical translation of these systems remains limited due to biological complexity, variability across tumor types, and challenges related to scalability, reproducibility, and regulatory approval.

In this context, protein-based nanomaterials represent a particularly promising class of nanoplatforms due to their intrinsic biocompatibility, biodegradability, and capacity for precise structural and functional engineering. Through a comparative and translational analysis, this review highlights that different protein-based systems occupy distinct positions along a spectrum of clinical maturity and functional complexity.

Established platforms such as human serum albumin demonstrate clear translational potential, supported by favorable pharmacokinetics, clinical precedent, and scalable production. In contrast, intermediate systems such as ferritin and transferrin offer advanced biological functionality but require further optimization to improve targeting specificity and manufacturing consistency. More complex platforms, including elastin-like polypeptides, virus-like particles, protein–polymer hybrids, and membrane-coated nanocarriers, provide high levels of multifunctionality and tunability, but currently face significant barriers related to immunogenicity, large-scale production, and regulatory feasibility.

From a future perspective, the field must move beyond the design of increasingly sophisticated nanosystems toward their effective clinical implementation, including dosing strategies and routes of administration. In particular, a shift toward systemic delivery approaches is required, together with a deeper understanding of the in vivo journey of these nanocarriers, from circulation to tumor accumulation and clearance. In this context, a critical step toward clinical translation will be the transition from largely artisanal, proof-of-concept formulations to standardized and scalable platforms through the engineering of robust bioproduction processes to ensure reproducibility, regulatory compliance, and successful clinical implementation. Moreover, biomedicine and, in particular, nanomedicine will be led by Artificial Intelligence (AI). Thus, the future of AI-assisted design for protein-based nanomaterials is moving from predictive modeling (structural forecasting) to autonomous, goal-directed generation. Over the next decade, AI will fundamentally change how polymers like ELPs and silk fibroins are conceptualized, optimized, and manufactured for clinical workflows. Current AI tools often specialize in either predicting 3D structure (i.e., AlphaFold) or generating sequences (i.e., ProtGPT2). The next generation of AI will feature multimodal models that directly bridge the gap between molecular sequence and macroscopic material properties. Ultimately, the future of protein-based nanomedicine will depend on the ability to balance biological sophistication with manufacturability, defining a new generation of clinically viable and scalable nanotherapeutic platforms.

## Figures and Tables

**Table 1:** Representative protein-based theranostic nanosystems. Platforms are categorized according to nanosystem type, functionality, and translational stage, highlighting the balance between multifunctionality and clinical feasibility.

Protein/System	Representative Nanosystem	Type of Nanosystem	Applications	Functional Advantage	Translational Stage	Refs.
Albumin (BSA)	Gd2O3/CuS-Cy7.	Hybrid protein nanocarrier	MRI/NIR imaging; PTT	Enables multimodal imaging-guided photothermal therapy	Preclinical	[150,151,152]
Human serum albumin (HSA)	Ag2S nanodots; phthaloNO2 NPs	Protein nanocarrier	NIR-II imaging; PTT/PDT	Clinical precedent; low immunogenicity	Advanced preclinical	[152,160,161,163,164,165,166]
Silk fibroin	ICG-SF nanoparticles	Protein–polymer nanocarrier	Fluorescence imaging; PTT	Structural versatility	Preclinical	[171,172,173,174]
Elastin-like polypeptides	Dox/PPy-ELP-F3	Stimuli-responsive nanoplatform	Imaging-guided chemo/PTT	Clinically relevant platform for imaging-guided therapy with low immunogenicity	Preclinical	[174,177]
Ferritin	Dox/Bi2S3	Protein nanocage	CT imaging; chemo/radiotherapy	Tunable polymer matrix enabling combined imaging and therapy	Advanced preclinical	[181]
Apoferritin (HFn)	MnO2-Dox@HFn; IR1061/PTX@AFN	Targeted nanocage/nanozyme	MRI-guided therapy; PTT + chemotherapy	Thermally triggered assembly enabling controlled drug release	Advanced preclinical	[184,185]
Transferrin	IR780-Tf NPs; Tf-Cu NCs/Dox	Ligand-targeted nanosystem	NIR imaging; PDT/PTT; FRET	Nanocage-enabled encapsulation for imaging-guided combination therapy	Preclinical	[187,188,189]
Virus-like particles (VLPs)	Qβ/P22 capsids	Protein cage nanoplatform	Drug delivery; MRI	Receptor-mediated uptake with pH-responsive controlled release	Early preclinical (oncology)	[191,192,193]
Protein–polymer hybrids	ROS-responsive nanoparticles	Hybrid nanosystem	PDT; imaging; immunotherapy	TfR-mediated targeting for imaging and intracellular delivery	Preclinical	[194]
Cell membrane-coated systems	RBC/platelet/tumor-coated NPs	Biomimetic nanosystem	PTT; chemo; immunotherapy	Modular capsid design enabling multivalent functionalization	Preclinical (high complexity)	[195,196,197,198,199]

**Table 2:** Quantitative comparison of protein-based theranostic nanosystems.

Protein System	Representative Platform	Tumor Inhibition (%)	Photothermal Efficiency (%)	Tumor-to-Normal Ratio	Circulation/Stability	Drug Loading (wt%)	Refs.
Albumin (HSA/BSA)	Gd_2_O_3_/CuS, IrO_2_-BSA, Ag_2_S-HSA	~100%	~30–45%	~5–10	Long (hours–days)	10–30	[106,107,108,109,201]
Ferritin (HFn/AFN)	MnO_2_-Dox@HFn, Bi_2_S_3_-AFN	up to 78.5%	~25–40%	~26.8 ± 4.1	Moderate	up to ~60	[146,147,148,149,150]
Transferrin (Tf)	IR780-Tf, Tf-Cu NCs/Dox	High (model-dependent)	~20–35%	~5–8	~48 h stability	5–20	[152,153,154]
Silk fibroin (SF)	ICG-SF, MnO_2_-SF-Dox	Moderate–high	~20–30%	~3–6	Moderate	10–25	[125,126,127,133]
Virus-like particles (VLPs)	Qβ, P22 capsids	Moderate	~15–30%	Target-dependent	Moderate	High (>50)	[156,157,158]
Protein–polymer hybrids	ROS-responsive NPs	High	~20–35%	~4–8	Good	10–40	[159]
Membrane-coated systems	RBC/platelet/tumor-coated NPs	High	~20–40%	~10–20	Very long	Variable	[160,161,162,163,164]

## Data Availability

No new data were created or analyzed in this study.

## Abbreviations

The following abbreviations are used in this manuscript:

AFN	Natural Apoferritin
BSA	Bovine Serum Albumin
BNCT	Boron Neutron Capture Therapy
ECM	Extracellular Matrix
ELPs	Elastin-Like Polypeptides
CPP	Cell-Penetrating Peptide
CRC	Colorectal Cancer Cells
CT	Computed Tomography
DLA	Dalton’s Lymphoma Ascites
Dox	Doxorubicin
EGFR	Epidermal Growth Factor Receptor
EMA	European Medicines Agency
FA	Folic Acid
FDA	United States Food and Drug Administration
FRα	Folate Receptor Alpha
GMP	Good Manufacturing Practices
HAS	Human Serum Albumin
HFn	Heavy-chain Apoferritin
HIV	Human Immunodeficiency Virus
HPV	Human papillomavirus
ICG	Indocyanine Green
IONPs	Iron Oxide Nanoparticles
GFLG	Lysosomal Enzymes
GFP	Green Fluorescent Protein
MRI	MRI
NFκB	Nuclear Factor kappa B
NIR	Near-Infrared
NPs	Nanoparticles
NSCLC	Non-Small-Cell Lung Cancer
PDT	Photodynamic Therapy
PGA	Poly(glycolic acid)
PLA	Polylactic Acid
PLGA	Poly(lactic-co-glycolic acid)
PPy	Polypyrrole
PTT	Photothermal Therapy
PTX	Paclitaxel
ROS	Reactive Oxygen Species
SF	Silk Fibroin
SPARC	Secreted Protein Acidic and Rich in Cysteine
Tf	Transferrin
TfR1	Transferrin Receptor 1
TLR9	Toll-Like Receptor 9
TME	Tumor Microenvironment
TmEnc	Thermotoga maritima encapsulin nanocages
VLPs	Virus-Like Particles
